# Neuroprotective effects of the PPARβ/δ antagonist GSK0660 in in vitro and in vivo Parkinson’s disease models

**DOI:** 10.1186/s40659-023-00438-1

**Published:** 2023-05-25

**Authors:** Andrea Antonosante, Vanessa Castelli, Martina Sette, Margherita Alfonsetti, Mariano Catanesi, Elisabetta Benedetti, Matteo Ardini, Annamaria Cimini, Michele d’Angelo

**Affiliations:** 1 Dpt of Life, Health and Environmental Sciences, University of L’Aquila, L’Aquila, Italy; 2 Sbarro Institute for Cancer Research and Molecular Medicine, Dpt of Biology, Temple University, Philadelphia, USA

**Keywords:** Parkinson’s disease, Oxidative stress, Proteasome, Mitochondrial impairment, Neurotrophic support, GSK0660

## Abstract

**Background:**

The underlying mechanism of Parkinson’s disease are still unidentified, but excitotoxicity, oxidative stress, and neuroinflammation are considered key actors. Proliferator activated receptors (PPARs) are transcription factors involved in the control of numerous pathways. Specifically, PPARβ/δ is recognized as an oxidative stress sensor, and we have previously reported that it plays a detrimental role in neurodegeneration.

**Methods:**

Basing on this concept, in this work, we tested the potential effects of a specific PPARβ/δ antagonist (GSK0660) in an in vitro model of Parkinson’s disease. Specifically, live-cell imaging, gene expression, Western blot, proteasome analyses, mitochondrial and bioenergetic studies were performed. Since we obtained promising results, we tested this antagonist in a 6-hydroxydopamine hemilesioned mouse model. In the animal model, behavioral tests, histological analysis, immunofluorescence and western blot of *substantia nigra* and *striatum* upon GSK0660 were assayed.

**Results:**

Our findings suggested that PPARβ/δ antagonist has neuroprotective potential due to neurotrophic support, anti-apoptotic and anti-oxidative effects paralleled to an amelioration of mitochondria and proteasome activity. These findings are strongly supported also by the siRNA results demonstrating that by silencing PPARβ/δ a significative rescue of the dopaminergic neurons was obtained, thus indicating an involvement of PPARβ/δ in PD’s pathogenesis. Interestingly, in the animal model, GSK0660 treatment confirmed neuroprotective effects observed in the in vitro studies. Neuroprotective effects were highlighted by the behavioural performance and apomorphine rotation tests amelioration and the reduction of dopaminergic neuronal loss. These data were also confirmed by imaging and western blotting, indeed, the tested compound decreased astrogliosis and activated microglia, concomitant with an upregulation of neuroprotective pathways.

**Conclusions:**

In summary, PPARβ/δ antagonist displayed neuroprotective activities against 6-hydroxydopamine detrimental effects both in vitro and in vivo models of Parkinson’s disease, suggesting that it may represent a novel therapeutic approach for this disorder.

**Supplementary Information:**

The online version contains supplementary material available at 10.1186/s40659-023-00438-1.

## Background

Parkinson’s disease (PD) is one of the most frequent brain disorders, involving about 1–4% of people older than 60 years [1]. The main symptoms of PD include motor impairment, since 60–80% of the dopaminergic neurons are lost [1]. The loss of dopaminergic neurons leads to dopamine decrease. Dopamine plays as a signal between two brain areas, the *substantia nigra* and the *corpus striatum*, which are responsible for controlled movements [2]. When dopamine levels are decreased, the communication between them is impaired, and the movement starts to be altered [3]. The underlying mechanisms of PD are still unclear, but it has been proposed the involvement of oxidative stress, excitotoxicity, and neuroinflammation. In particular, free radical injury, resulting from oxidative dopamine metabolism by monoamine oxidase induces to the formation of hydrogen peroxide, which is usually eliminated by catalase and glutathione, but, if not effectively removed, it may lead high levels of reactive hydroxyl radicals able to react with cellular components, resulting in lipid peroxidation and cell damage [4, 5]. The pathogenesis of neurodegenerative diseases, including PD has also been ascribed to decreased neurotrophic support [6]. Several findings reported on the relevance of neurotrophic factors for neurons’ survival [7]. A reduced ratio between mature brain-derived neurotrophic factor (mBDNF) and its immature form (pro-BDNF) was detected during normal aging and neurodegenerative disorders [8, 9]. Pro-BDNF interacts with the pan-neurotrophic receptor p75NTR leading, together with sortilin, to neuronal death [10], with a concomitant upregulation of the truncated form of Tropomyosin receptor kinase B (TrkB) and a reduction of the full-length TrkB (TrkBFl) [11].

We have previously reported that the peroxisomal PPARβ/δ is implicated in the reduction of the TrkBFl, mainly in neurodegeneration [12–14]. PPARs are a class of transcription factors expressed throughout the body, including three subtypes, PPARα, PPARβ/δ, and PPARγ. PPARβ/δ is most copious at brain level, followed by PPARγ and then PPARα [15]. PPARs control gene expression by heterodimerizing with retinoid X receptors [16]. The resultant complex interacts to promoter sequences of target genes [16, 17], comprising those mediating differentiation, energy metabolism, inflammation, oxidative stress, cell growth, and neurodegeneration [12]. Undoubtedly, the role of PPARγ in neurodegenerative disorders is the most studied, especially regarding Pioglitazone and other agonists, showing protective effects on injured brain [18–21], including PD. In this study we decided to focused on PPARβ/δ, which is an oxidative stress sensor, being specifically activated by 4-Hydroxynonenal (4-HNE) [22]. In addition, in our previous work, in the brain of hemilesioned PD animals, the increase of PPARβ/δ was paralleled by a rise in pro-BDNF and activation of death pathways [23], thus suggesting the potential use of a PPARβ/δ antagonist in counteracting primary and possibly secondary PD symptoms.

On these bases, in the present work, the molecular pathways activated were dissected in an in vitro model of PD together with the effects induced by a specific PPARβ/δ antagonist, GSK0660. The promising results obtained in the in vitro model were implemented with in vivo experiments performed on a 6-hydroxydopamine (6-OHDA) hemilesioned PD mouse model treated with GSK0660. The data obtained in this study confirmed our previous findings on the detrimental role of PPARβ/δ in oxidative stress-mediated neurodegeneration, demonstrating the positive effects of the antagonist. Overall, GSK0660 displayed neuroprotective activities against 6-OHDA-induced injury both in vitro and in vivo, suggesting that it may represent a novel therapeutic approach for PD.

## Materials and methods

### In vitro**model**

#### Cellular model

As cellular model, human neuroblastoma cell line SH-SY5Y was used (ATCC # CRL-2266) and cultured following manufacturer’s protocols in Dulbecco’s minimum essential medium (DMEM) complemented with 10% heat-inactivated fetal bovine serum (FBS), and 1% L-glutamine at 37 °C (95% air and 5% CO2). All experiments were made at the same passage and the cell culture was routinely tested for Mycoplasma (Mycoplasma PCR, ABM, USA).

For obtaining dopaminergic-like phenotype, cells were cultured under differentiation conditions. 24 h after plating (1.5 × 10^4^ cells/cm^2^), cells were grown in culture media supplemented with 1% FBS and 10µM All-Trans Retinoic acid (ATRA) for three days. The media was then removed and replaced with culture medium supplemented with 1% FBS and 80nM 12-O-tetrade-canoyl-phorbol-13-acetate (TPA) and maintained in these culture conditions for another three days. For the setup of PD model, 6-OHDA was added in the dopaminergic-like differentiated cells (see the following paragraph).

#### Treatments

Six days after differentiation, the dopaminergic-like in vitro model was exposed to culture conditions that mimic some PD mechanisms. The cells were exposed to different concentrations of 6-OHDA neurotoxin in culture media supplemented with 1% FBS for 24 h. 6-OHDA 25 µM induced about a 40% of cell death; thus, this concentration was selected to perform subsequent experiments. To study the effects induced by a PPAR β/δ antagonist (GSK0660, Sigma Aldrich, Saint Louis, USA) in our PD in vitro model, cells were co-treated with GSK0660 0.2 µM and 6-OHDA 25 µM. The in vitro model was exposed to GSK0660 in culture media supplemented with 1% FBS, 30 min later, 6-OHDA 25µM (diluted in culture media with 1% FBS) was added. Our in vitro model was maintained in these culture conditions for 24 h. Both 6-OHDA and GSK0660 stock solutions were prepared in Dimethyl sulfoxide and maintained at -20° C.

#### Cell viability

To analyze the cell viability upon treatments, Cell Titer One Solution Cell Proliferation Assay (MTS) (Promega Corporation Madison, WI, USA) was performed. The quantity of formazan produced was read at 490 nm in a microplate reader, Infinite F200 (Tecan, Männedorf, Swiss). In the first series of experiments, cells were treated with different concentrations of 6-OHDA (100, 75, 50, and 25 µM) to evaluate the optimal condition to perform the co-treatment with GSK0660. In the second series of experiments, cells were exposed to GSK0660 0.2 µM and 30 min later, they were stressed with 6-OHDA 25 µM for 24 h. Results were expressed as cell viability (% vs. control). The value of untreated cells was considered 1 intended as 100% of cell viability.

#### Cell index real-time analysis

The effects of 6-OHDA and GSK0660 on our in vitro model were evaluated by Cell Index (CI) Real-time analysis using an xCELLigence Real-Time Cell Analyzer (RTCA) DP system (ACEA Biosciences, San Diego, CA, USA) at 37 °C in 5% CO2. The baseline was determined adding 100 µL of culture media to each well of an E-Plate 16, and the baseline value for each well in the plate was recorded. Then, the cells were seeded at a density of 1.5 × 10^4^ cells/well into the E-plate wells and differentiated as described above. When cells reached the log phase, GSK0660 was added to a final concentration of 0.2 µM, 30 min later, 6-OHDA was added to a final concentration of 25 µM. The cell response was continuously monitored for 24 h.

The xCELLigence system gives a quantitative parameter named CI, reflecting the cell status. In brief, this system methods cell-electrode impedance; thus, the CI is a measure of cell number, cell viability, adhesion degree. The results are reported as Normalized Cell Index (NCI) for each well.

#### IncuCyte caspase-3/7 green and cytotox green

Differentiated cells were seeded (10^4^ cells/cm^2^) into a black 96 wells plate. Cells were then exposed to the treatments as described above, and 250 nM of IncuCyte Cytotox Green Reagent (Essen BioScience, USA) were used to evaluate dead cells. To determine apoptosis, 5 µM IncuCyte Caspase-3/7 Green (Essen BioScience) were used. Plate was put in IncuCyte device (20x objective), and caspases activation and cytotoxicity were performed (3 images for well) every 3- 24 h (final timepoint). Images were analysed applying the Incucyte ZOOM software, and data were described as green object count per mm^2^.

#### Western blotting

Cells were treated as described above. Lysates from each sample were obtained by radioimmunoprecipitation assay buffer RIPA lysis buffer (Thermo Scientific, USA) supplemented with freshly added protease and phosphatase inhibitors. After, lysates were kept on ice for 30 min. Total proteins were extracted by centrifugation at 14,000 rpm for 30 min at 4 °C. Protein concentration was evaluated using Bicinchoninic acid (BCA) assay (Thermo Scientific, USA). 20/30µg of protein were run on 4–12% Tris-Glycine gels and then blot onto polyvinylidene difluoride (PVDF) membranes using semidry system (Thermo Scientific, USA). Then membranes were washed in 1x TBS-T (tris-buffered saline with 0.05% Tween-20, pH 8) and blocked in 5% not fat milk for 1 h at RT. Membranes were then incubated with the following primary antibodies: anti-pAKT (Ser473) (1:1000), anti-PI3K p110 (1:1000), anti-Tyrosine hydroxylase (TH) (1:1000), anti pCREB (Ser133) (1:500), anti-pTrkBFL (Tyr515) (1:500), anti-mBDNF (1:500), anti-4-HNE (1:500), anti-cleaved Caspase-3 (1:1000), anti-cleaved Caspase-9 (1:500), anti-PPARβ/δ (1:1000), anti-cleaved PARP (1:1000), anti-Parkin (1:500), anti-DJ-1 (1:500), anti OPA1 (1:), anti Mfn (1:), anti Drp1 (1:) and anti-pBcl2 (Ser70) (1:1000) overnight at 4 °C. After washes in TBS-T, membranes were incubated with 1:20000 HRP-conjugated anti-rabbit IgG or anti-mouse IgG. Anti-β-actin (HRP-conjugate) (1:10000, Cell Signaling, USA) was used as a loading control. Immunoreactive protein bands were visualized by with West Pico luminol (Thermo Scientific), according to the manufacturer’s protocol using Alliance Q9 (Uvitec, UK). The results were analyzed by ImageJ software and normalized to β-actin, and values were given as relative units (R.U.).

#### Subcellular protein fractionation kit

For the subcellular protein fractionation, the kit #78,840, Thermo Scientific, USA was used. Cells were cultured as described above, collected and incubated with 1000 µl ice cold CEB (cytoplasmatic extraction buffer) for 10 min at 4 °C with gentle mixing and then centrifugated at 500 g for 5 min. The supernatant (cytoplasmic extract) was put into a clean pre-chilled tube. 1000 µl of ice-cold MEB (membrane extraction buffer) were added to the cytoplasmic extract for 10 min at 4 °C and then tubes were centrifugated at 3000 g for 5 min. Supernatants (membrane extract) were put into a clean tube on ice. 1000 µl of ice-cold NEB (nuclear extraction buffer) were added in the tube with membrane extract and incubated at 4 °C for 30 min with gentle mixing. At this point, pellets were centrifuged at 5000 g for 5 min and supernatants (soluble nuclear extract) were transferred into different tubes. 5µL of 100mM CaCl_2_ and 3 µL of Micrococcal Nuclease were added to soluble nuclear extract and incubated at RT for 5 min. After incubation, the soluble nuclear extract was centrifuged at 16,000 g for 5 min. Supernatants were transferred into a clean pre-chilled tube. The protein concentrations for cytoplasmic extract and soluble nuclear extract were assayed by the Pierce BCA Protein Assay kit (Thermo Scientific) and diluted in sample buffer and then performed the Western blotting analyses as described above.

#### Measurement of cellular reactive oxygen species (ROS)

To determine ROS production in the in vitro model, 2′–7′-dichlorofluorescein diacetate (DCFDA) assay (Abcam, UK) was used according to manufacturer’s protocols. Cells were cultured and as described and then washed and incubated with 10 µM DCFDA for 30 min at 37 °C at the dark. After washes with Phosphate Buffered Saline (PBS), cells were treated as described above in medium w/o phenol red. H_2_O_2_ 800 µM was utilized as a positive control. ROS production was immediately measured by detecting the fluorescent dichlorofluorescein (DCF), using Tecan Spark (Tecan, Swiss), at an Ex-485 and Em-535 nm. Measurements were performed every 30 min for six hours. Values reported were acquired by the ratio of fluorescence at a specific time point on fluorescence at time 0, which was determined after DCFDA incubation.

#### Superoxide dismutase activity assay

The total Superoxide dismutase (SOD) activity was calculated using a SOD assay kit from Abcam, UK following manufacturer’s protocol. SOD activity was assessed by measuring the rate of reduction of WST-1, which creates a water-soluble formazan dye upon reduction with a superoxide anion. The absorbance of WST-1 was determined using Tecan Spark (Tecan, Swiss) at 450 nm.

#### Immunofluorescence

Cells were fixed and permeabilized in cold methanol for 10 min and then blocked with 4% Bovine serum albumin (BSA) for 30 min at RT and incubated O/N at 4° C with the subsequent primary antibodies: rabbit polyclonal anti-PPARβ/δ (1:500, Thermo Scientific, USA). After numerous washes, secondary antibodies, goat anti-mouse IgG Alexa Fluor 488 conjugated (1:2000 Life Technologies, USA) was used for 1 h at RT. After other washes, coverslips were mounted with Vectashield with 4′,6-diamidino-2-phenylindole (DAPI) (Vector Laboratories Burlingame, USA) and then examined at confocal laser microscope (Leica, Germany).

#### MitoTracker deep red and quantification of mitochondrial fragmentation

MitoTracker stains mitochondria fluorescently in live-cells, and its accumulation is due to membrane potential. Briefly, cells were cultured and differentiated on coverslips and treated as described above. MitoTracker deep Red (Invitrogen, USA) in Hanks’ Balanced Salt Solution (HBSS) buffer at concentration of 1 µM for 15 min was added. After washes, cells were fixed with 3.7% formaldehyde in PBS for 10 min at room temperature. Finally, coverslips were mounted with Vectashield with DAPI. Images were acquired using Leica TCS SP5 (Leica, Germany). Quantitative analysis of mitochondrial fragmentation was executed as previously reported [24, 25]. Mitochondria shorter than 2 μm were deemed fragmented, while longer than 5 μm were indicated as filamental. Images were binarized by the threshold module using ImageJ, and then converted to images 1 pixel wide by the skeletonize module. All the analyses were completed by a researcher blind to the experiment. Each experiment was repeated at least three times, and 16–25 cells per condition were quantified.

#### Oxyblot

OxyBlot kit (Merck Millipore, Burlington, MA, USA) was performed according to the manufacturer’s instructions to evaluate oxidized protein levels. In brief, cells were plated (density 1.5 × 10^4^ cells/cm^2^) in 10% FBS-supplemented medium. 24 h after seeding, the cells were exposed to our differentiation conditions, as previously mentioned. After complete differentiation, cells were exposed for 24 h to co-treatment with GSK0660 0.2µM, and 25µM 6-OHDA in culture media supplemented with FBS 1%, as previously described. Consequently, pellets were collected and homogenized in lysis buffer having dithiothreitol (DTT) 50mM. 5 µg/µl of protein extracts were derivatized following manufacturer’s protocols. 20 µg total proteins per sample were separated on 10% polyacrylamide SDS denaturing gels. As primary antibody rabbit anti-DNP diluted 1:150 was incubated. According to manufacturer’s instructions, after incubation with secondary HRP-conjugated anti-rabbit IgG antibody diluted 1:300, the immunoreactive bands were detected with West Pico luminol (Thermo Scientific). Bands were visualized using Alliance Q9 (Uvitec, UK) and analyzed by FIJI software. As housekeeping protein actin was used, and values were given as relative units (R.U.).

#### TaqMan gene array

TaqMan gene expression array cards (96-well plate format, which enables analysis of 2 samples against 48 genes) were custom made at Applied Biosystems. The list of genes examined in the array in Table 1 are reported. Total RNA was extracted by Trizol reagent (Thermo Scientific, USA), and RNA was quantified using Qubit Assay Kits (Thermo Scientific, USA) and RNA quality was confirmed by NanoDrop (Thermo Scientific, USA). 1 µg of total RNA was reverse transcribed into cDNA using ProtoScript First Strand cDNA Synthesis Kit (New England BioLabs). Taqman qRT-PCR was performed on ABI 7300HT (Applied Biosystems, USA) using the following thermal cycling conditions: 50 °C for 2 min; 95 °C for 10 min then 40 cycles at 95 °C for 15 s and 60 °C for 1 min. 10 ng cDNA samples mixed with 2× TaqMan Universal PCR Master Mix (Thermo Scientific, USA) were used. In parallel, to quantify the expression of BDNF and CREB in Small interference RNA studies the same protocol was used. Relative expression levels were determined for each sample after normalization against reference gene, using the ΔΔCt method for comparison of relative fold expression variations, as reported by [26]. For the Gene Ontology, the examination for biologically relevant functions was evaluated with Panther v.13.1 database.

**Table 1:** Fold changes of examined genes using TaqMan gene expression array cards

		FOLD vs CTR (2-^DDCT^)		
	6OHDA		6-OHDA + GSK0660	
**ATP2B2**	0,40	0,27	0,59	0,57
**BCL2**	0,77	0,68	0,77	1,08
**BDNF**	0,24	0,20	0,45	0,54
**CAT**	0,19	0,19	0,25	0,15
**CHGB**	0,41	0,60	0,63	0,86
**CREB1**	0,78	0,69	1,23	1,33
**CXXC1**	0,50	0,52	0,79	0,79
**DDC**	0,16	0,13	0,28	0,23
**DRD2**	0,48	0,45	0,50	0,59
**FBXO9**	0,44	0,42	0,78	0,74
**FGF13**	1,68	1,29	0,59	0,65
**GPR37**	0,25	0,33	0,44	0,43
**GPX1**	0,67	0,66	0,86	0,94
**ILK**	0,23	0,25	0,40	0,52
**LRRK2**	0,09	0,10	0,29	0,31
**MAPK9**	0,51	0,40	0,76	0,76
**MTOR**	0,50	0,53	0,78	0,72
**NCOA1**	0,37	0,39	0,69	0,84
**NFASC**	0,72	0,56	1,30	1,20
**NFE2L2**	0,65	0,74	1,03	1,29
**NGF**	2,66	1,56	3,05	2,44
**NR4A2**		0,79	1,27	1,81
**NSG1**	0,29	0,38	0,56	0,56
**NTRK2**	0,35	0,26	0,96	0,96
**OPA1**	0,48	0,43	0,79	0,82
**PARK2**	0,49	0,43	1,00	1,05
**PARK7**	0,41	0,37	0,80	0,82
**PINK1**	0,62	0,50	1,46	1,46
**PPARA**	0,79	0,67	1,06	1,19
**PPARGC1A**	0,67	0,64	1,08	1,20
**PPARG**	0,54	1,38	0,95	1,38
**PRDX2**	0,38	0,40	0,61	0,59
**RTN1**	0,33	0,29	0,45	0,50
**SLC25A4**	0,48	0,44	0,64	0,87
**SNCA**	0,47	0,48	0,72	0,74
**TBP**	0,49	0,54	0,80	0,78
**UBA1**	0,28	0,35	0,48	0,51
**UCHL1**	0,79	0,81	1,20	1,25
**VDAC3**	0,48	0,51	0,97	0,90

#### MitoSOX assay

To detect the presence of mitochondrial superoxide, MitoSOX assay was performed. MitoSOX Red Mitochondrial Superoxide is a fluorogenic dye used to detect superoxide in the mitochondria of living cells. Briefly, cells were seeded (cell density 1.5 × 10^4^ cells/cm^2^) in a black 96 wells plate, differentiated, and treated as described above. Then, cells were incubated with 5 µM of MitoSOX reagent solution, avoiding light exposure. IncuCyte device was used (20x objective), and relative fluorescence intensity analyzed by Incucyte ZOOM software was reported as normalized fluorescence.

#### mPTP assay and mitochondrial membrane potential (MMP)

To assess the permeability of transition pore in the inner mitochondrial membrane in our in vitro model Proteasome Mitochondrial PT Pore assay kit (Cayman Chemical, USA) Tetramethylrhodamine, ethyl ester (TMRE) staining for the measurement of MMP were used following manufacturer’s protocols as previously described [27]. Briefly, cells were plated on coverslips (cell density 1.5 × 10^4^ cells/cm^2^), differentiated, and treated as described above. Then coverslips were observed at confocal microscopy. Fluorescence intensity was examined by FIJI software and reported as normalized fluorescence.

#### Proteasome (chymotrypsin-like, trypsin-like, and caspase-like) activities assay

To evaluate chymotrypsin-like [Suc-LLVY-AMC] from Sigma Aldrich, trypsin-like [Boc-LRR-AMC] and caspase-like [Z-LLE-AMC] both from R&D System activities of the proteasome in our PD in vitro model were analyzed as previously described [27]. Briefly, cells were seeded (seeding density 1.5 * 10^4^ cells/cm^2^) in culture medium, differentiated, and treated as described above GSK0660 0.2µM and 6-OHDA 25µM treatments were diluted in culture media w/o phenol red supplemented with FBS 1%, as described above. After treatments, cells were trypsinized and centrifuged 6 min at 250 *g*. For each experiment, the time interval considered was 30 min. µg of the protein was the total amount of the protein in the well. The results are reported as ΔRFU/min/µg of protein.

#### Characterization of proteasome

Proteasome structures were characterized by native gel electrophoresis/western blotting (5%), as previously reported [28, 29]. In brief, cells were plated (seeding density 1.5 * 10^4^ cells/cm^2^) in 10% FBS-supplemented medium. 24 h after seeding, the cells were exposed to our differentiation conditions, as previously mentioned. upon complete differentiation, the cells were exposed to co-treatment with GSK0660 0.2µM, and 6-OHDA 25µM for 24 h in culture media supplemented with FBS 1%, as described above. Then, pellets were homogenized in lysis buffer as previously described [27]. Samples were prepared 1:1 in native sample buffer (BioRad, USA) and loaded in native gel conditions. The running gel was made of resolving gel (5%) with freshly added 1 mM ATP and then run in TBE buffer at 50 V for 40 min, 100 V for 30 min and 150 V for 3 h, then transferred onto PVDF membranes (Thermo Scientific, Rockford, IL, USA) through wet electrophoretic transfer (constant 400 mA for 2 h and 30 min at + 4 °C). After blocking with 5% skimmed milk, membranes were incubated with rabbit anti-human PSMA6 (1:5000) (Abcam, UK), followed by HRP anti-rabbit IgG antibodies incubation (1:10000).

#### Seahorse mito stress assay

Seahorse XF96e Extracellular Flux Analyzer (Agilent Technologies, CA, USA) to analyze the bioenergetic profiles of our PD in vitro model upon treatments was employed. Live-cell evaluations of oxygen consumption rate (OCR) and extracellular acidification rate (ECAR) were evaluated utilizing the Mito Stress assay (Agilent, USA). Cells were seeded on a Seahorse XF96 plates at a density of 5 * 10^4^ cells/well (optimized to guarantee a proportional response of FCCP) then differentiated and treated as explained above. Mito Stress assay was performed following manufacturer’s protocols and the concentrations of the drugs were: 1 µ*M* oligomycin, 1 µ*M* FCCP, 0.5 µ*M* rotenone/antimycin. For the normalization in port D, Hoechst 33,342 solution was added. Data were obtained using Wave software.

#### Small interference RNA (siRNA)

According to the manufacturer’s instructions, gene silencing by siRNA was induced by transfecting cells with target-specific siRNA for PPARβ/δ or scrambled sequence, at the final concentration of 25 nM. Briefly, after RA/TPA differentiation, the media was removed and replaced with DMEM 1% FBS, 1% glutamine, and no antibiotics. The transfection complex was performed as follows: target-specific siRNA for PPARβ/δ (Ambion, Austin, TX, USA) or scrambled sequence were diluted into media and mixed with the transfection agent (Transit X2 by Mirus Bio LLC, USA). The mixture was incubated for 30 min at room temperature to let the transfection complex generation. This complex was then put into the well, and cell suspension was incubated for 72 h at 37 °C. siRNA scrambled sequence was used as control.

### In vivo experiments

#### Animals

Animal handling and surgical processes were done to minimize discomfort and pain, basing on the ethical regulations of the European Communities Council (Directive 2010/63/EU, protocol #542/2019-PR).

As animal model 9-week-old male C57BL/6 were used (Charles River, Massachusetts, USA). Mice (n = 36) were maintained in ventilated cages (Tecniplast, Germany) under a 12-hour light/12-hour dark cycle with food and water always available. Stereotaxic (Stoelthing, USA) injections of 6-OHDA were executed as described before [30]. In brief, animals were anesthetized (xylazine (10 mg/kg) and ketamine (200 mg/kg)) and then 4 µg of 6-OHDA with 0.2% ascorbic acid (volume: 2 µl, Sigma Aldrich, USA) in saline solution or vehicle only (SHAM group) were injected into the right region of the *striatum* (coordinates in relation to bregma: medial-lateral + 0.18 cm; anteroposterior + 0.04 cm; dorsal-ventral + 0.35 cm) with a rate of 0.5 µl/minute by means of single syringe nano Infusion KDS 310 (KD Scientific, USA) (5 min before removal of the syringe were waited).

#### Treatments

GSK0660 solution was freshly prepared, and the treated mice received 1 mg/kg via intraperitoneal injection. GSK0660 was administered 1 day previous 6-OHDA injection and for additional 3 weeks (daily). Control group received GSK0660 only.

#### Behavioral tests

We performed both drug-induced (apomorphine-induced rotation) and spontaneous motor tests (cylinder, elevated body swing), which are reliable and sensitive tests in unilateral 6-OHDA animal models. Investigators were blinded to the treatment conditions and before performing the different tests, animal were allowed to habituate for at least 10 min before evaluation.

#### Cylinder test

Cylinder rearing test [31] was adapted for use in mice to study forelimb usage in spontaneous behaviour. The cylinder test is a common behavioral assessment utilized for evaluating the motor impairments in experimental models of Parkinson’s disease, including unilateral injection of 6-OHDA. Hemilesioned animals showed a strong forelimb asymmetry due to lesion of the nigrostriatal pathway [32]. While a healthy mouse uses the right and the left paw with no preference, dopamine-lesioned mice show preferential use of the paw ipsilateral to the lesion. Each mouse was placed in cylinder as previously described [30]. Forelimb contacts were documented (20 contacts). Paired and impaired forelimb contacts were calculated as % of contralateral paw use recorded during the observation time. All the behavioural tests were performed the day before the sacrifice of the animals.

#### Elevated body swing test

Another sensitive test for the evaluation of motor asymmetry in the unilateral model of PD is elevated body swing test (EBST) [33, 34]. To perform EBST, animals were gently picked up at the base of the tail and the swings (left or right) were registered until 20 swings as previously reported [35].

#### Apomorphine test

Apomorphine-induced rotation test was executed to evaluate the *striatum* lesion. Animals were placed in a cylinder with a diameter of 11.5 cm and height of 14 cm, in a closed room to avoid any environmental disturbance, and allowed to habituate for at least 10 min before injection with a specific dose of drug. Specifically, 0.1 mg/kg of apomorphine (dissolved in a 0.2 mg/mL ascorbic acid in 0.9% saline solution) were subcutaneously administered in each mouse and thus observed for 40 min. Data were reported as contralateral rotation per min.

#### Morphological analysis

Mice were anesthetized and then sacrificed by cardiac perfusion with PBS, followed by 4% PFA in 0.12 M phosphate buffer, pH 7.6. Then, brains were collected and left O/N in 4% PFA, then transferred in 30% sucrose solution. Specimens were embedded in the OCT (Thermo Scientific, USA) and then cut using a cryostat to obtain coronal 40 μm thick sections as previously reported [30].

#### Immunohistochemistry

Free floating sections were incubated in 4% BSA for 1 h at RT and then incubated O/N at 4 °C with rabbit polyclonal anti-TH (1:500). In control sections, primary antibody was omitted. For blocking internal peroxidases 0.3% hydrogen peroxide solution for 10 min was used. After washes sections were incubated for 2 h at RT with HRP-conjugated goat anti-rabbit (1:100, Sigma, USA) in PBS 0.4% Triton X100. 3,3′-diamino-benzidine (DAB Substrate Kit for Peroxidase, Vector) was used as the chromogen. Sections were washed, and mounted with Eukitt® Quick-hardening mounting medium (Sigma, USA) and observed with a Leica S5. To quantify TH + cells, 3 slices were used *per* each group (n = 3).

#### Immunofluorescence

Free floating sections were treated as described in “immunohistochemistry” paragraph and incubated O/N at 4 °C with primary antibodies: chicken polyclonal anti-TH (1:500), mouse anti-Iba1 (1:500) and rabbit anti-GFAP (1:500). Specimens were washed and incubated for 2 h at RT with secondary antibodies Alexafluor 488 conjugated donkey anti-chicken IgG 1:1000 and Alexa633 conjugated donkey anti-mouse IgG 1:1000 and Alexa549 conjugated anti-rabbit IgG 1:1000 (Thermo, USA). Controls were performed by omitting the primary antibody. Images were acquired using Leica TCS SP5 confocal microscope and analysed by ImageJ software. For GFAP and Iba1 fluorescence intensity quantification, Fiji software was employed and data reported as fluorescence intensity % of control, evaluating 9 different fields for each condition (*n* = 3 animals each group; 3 fields *per* animal).

#### Western blot

Under stereomicroscope, *substantia nigra* and *striatum* were isolated and lysate using pestles to extract and quantify proteins as reported by [30]. 20 µg of proteins were run on 4–13% Nupage Bis-Tris precast gel (Thermo Scientific, USA) in running buffer at 200 V for 1 h, then transferred onto PVDF membranes using a semi-dry device (Thermo Scientific, USA) for 10 min in 1 Step buffer. Membranes were incubated with Blocking Buffer (Thermo Scientific, USA) for 15 min at RT and then incubated O/N at 4 °C with the following primary antibodies: anti-TH (1:1000), anti-pCreb (Ser133) (1:500), anti pAKT (Ser473) (1:1000) and anti-mBNDF (1:1000), diluted in the same blocking solution: at 4 °C O/N and then incubated with 1:10000 HRP-conjugated secondary antibody. Protein bands were detected with West Pico luminol (Thermo scientific) using Alliance Q9 (Uvitec, UK). For the densitometric analyses, ImageJ was used and normalized upon the housekeeping protein (HRP-conjugated actin).

### Statistical analysis

Data are mean ± SE of 3 separate experiments. one-way ANOVA followed by Tukey’s multiple comparisons test were used using Graphpad Prism 9. In the case of time lapse analysis, two-way ANOVA was used. p < 0.05 was set as level of significance.

## Results

### Cell viability assays in PD in vitro model

**Fig. 1:**
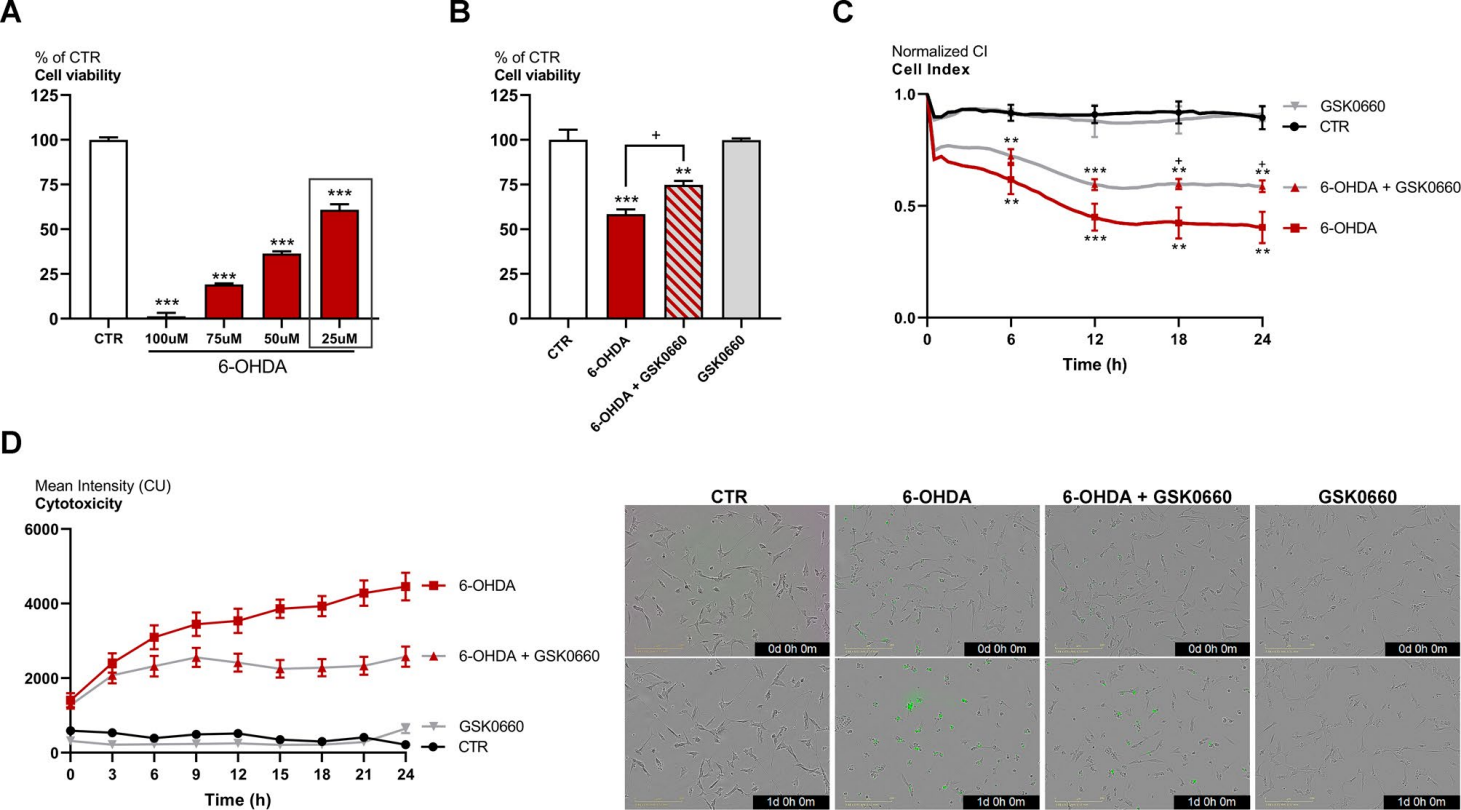
(**A**) MTS assay of differentiated SH-SY5Y treated with different concentration of 6-OHDA for 24 h. (**B**) MTS assay of cells treated with 25 µM 6-OHDA and 25 µM 6-OHDA + 0.2µM GSK0660. (**C**) Delta Cell index (DCI) evaluation in control and treated cells. (**D**) Live-cell IncuCyte cytotoxicity assay in cell treated with 25 µM 6-OHDA, 25 µM 6-OHDA + 0.2µM GSK0660 and 0.2µM GSK0660 alone. A representative figure for CTR and treated cells at 0 and 24 h is shown (n = 3). Scale bar: 200 μm. *** p < 0.0005; ** p < 0.005; * p < 0.05 vs. control (CTR). ^+^p < 0.05 vs. 6-OHDA (n = 3)

The in vitro model used in this study of SH-SY5Y-induced dopaminergic phenotype was developed and characterized as already described [30]. PD cellular model was obtained by 6-OHDA challenge (Fig. 1A). MTS assay revealed that the concentration of 6-OHDA able to lead a significant reduction in cell viability was 25 µM and thus selected for the following experiments (Fig. 1A). In Fig. 1B, the MTS assay of cells treated with 25 µM 6-OHDA alone or in combination with the PPARβ/δ antagonist GSK0660 is shown. GSK0660, administered at 0.2 µM was able to significantly counteract the 6-OHDA-induced mortality. To further support these data, cell index analysis paralleled with cytotoxicity live-imaging assay were performed (Fig. 1C **and D**). The results obtained confirmed the cell viability assay showing that 6-OHDA challenge determined a significant decrease in Cell Index and an increase of cytotoxicity, while GSK0660 showed neuroprotective effects.

### PPARβ/δ localization and oxidative stress analyses in the in vitro model

**Fig. 2:**
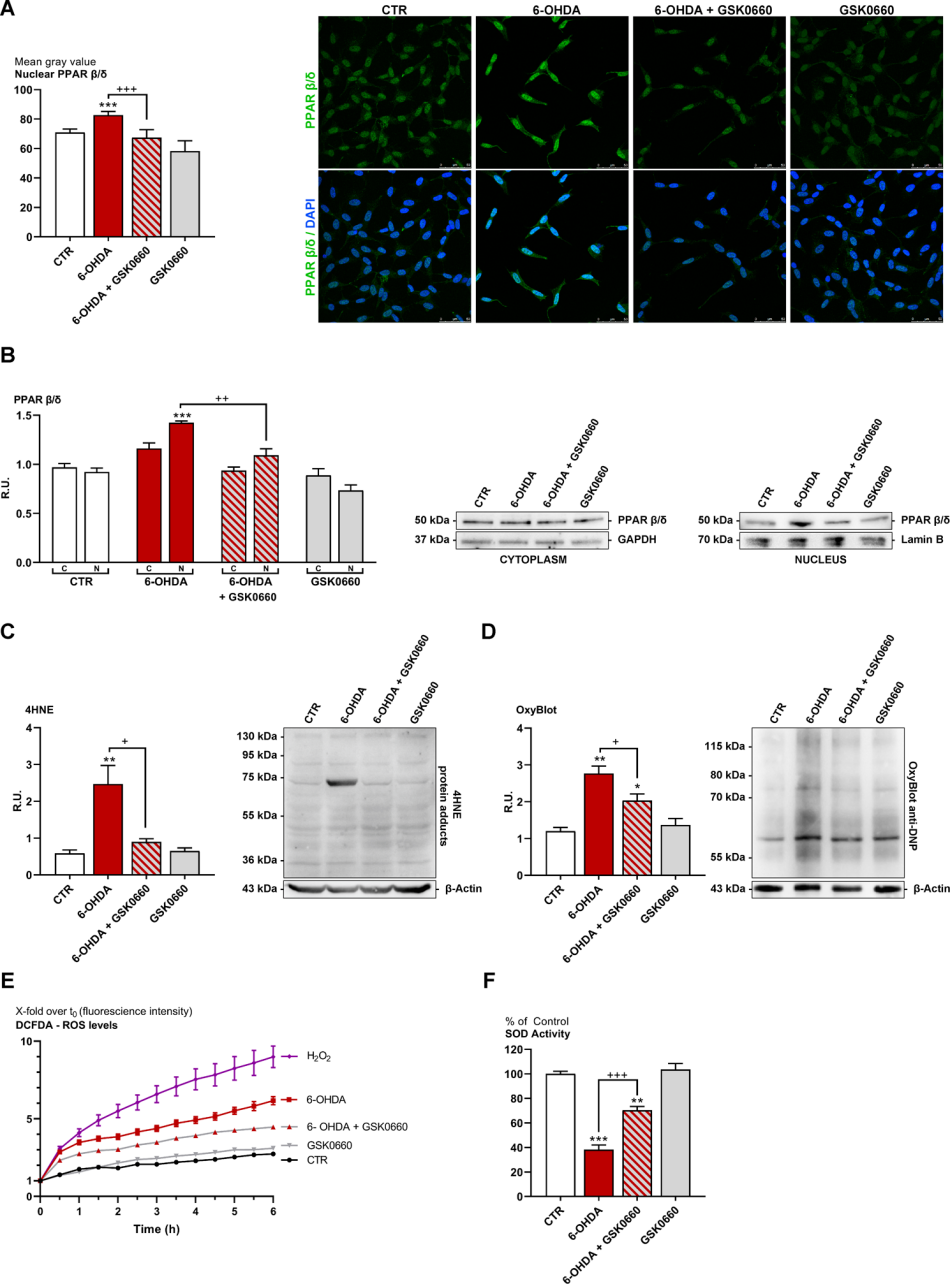
(**A**) Immunofluorescence representative images and relative quantification for the marker PPAR β/δ (in green) in control and treated cells. (In blue DAPI). Scale bar: 50 μm. (**B**) WB and relative densitometric analysis for cytoplasmic and nuclear PPAR β/δ in control and treated cells. (**C**) WB and relative densitometric analysis for control and treated cells for 4-HNE proteins adducts. (**D**) Oxyblot representative image of the membrane and relative densitometric analysis. (**E**) ROS level evaluation (DCFDA assay) in control and treated cells. (**F**) SOD activity in control and treated cells. *** p < 0.0005; ** p < 0.005; * p < 0.05 vs. control (CTR). ^+++^p < 0.0005; ++p < 0.005; +p < 0.05 vs. 6-OHDA (n = 3)

In Fig. 2A and 2B, PPARβ/δ nuclear localization assayed by immunofluorescence and western blotting analyses in control, 6-OHDA, 6-OHDA + GSK0660 and GSK0660 treated cells is reported. It is possible to observe that in control neurons, the transcription factor is weakly present, while upon 6-OHDA challenge this factor resulted increased, showing nuclear localization. The presence of the antagonist in the combined treatment restored the control condition (Fig. 2A and 2B).

Since it is known that the oxidative stress marker 4-HNE activates PPARβ/δ and that it is a marker of lipid peroxidation, the protein adducts of this compound were assayed by Western blotting analysis. (Fig. 2C). It is possible to observe, upon 6-OHDA, a significant increase of HNE-protein adducts at ≃72 kDa, whereas the presence of the antagonist restored to control levels. In the same figure (Fig. 2D), the Oxyblot analysis showing the levels of oxidized proteins in control and treated cells is reported. 6-OHDA significantly increased oxidized proteins, while the presence of the antagonist restored the control conditions. In Fig. 2E, ROS levels measured by DCFDA assay were shown. Starting from 1 h, 6-OHDA and H_2_O_2_ (positive control) showed an upward trend of ROS levels compared to untreated cells, while GSK0660 in combination with 6-OHDA counteracted this effect. Moreover, as shown in the same figure (Fig. 2F), the 6-OHDA challenge caused a significant decrease in SOD activity. Notably, the presence of the antagonist significantly increased the activity of SOD. Taken together this data indicated that GSK0660 treatment significantly counteracted oxidative stress due to the detrimental effect of 6-OHDA.

### Analyses of survival and apoptotic pathway in the in vitro model

**Fig. 3:**
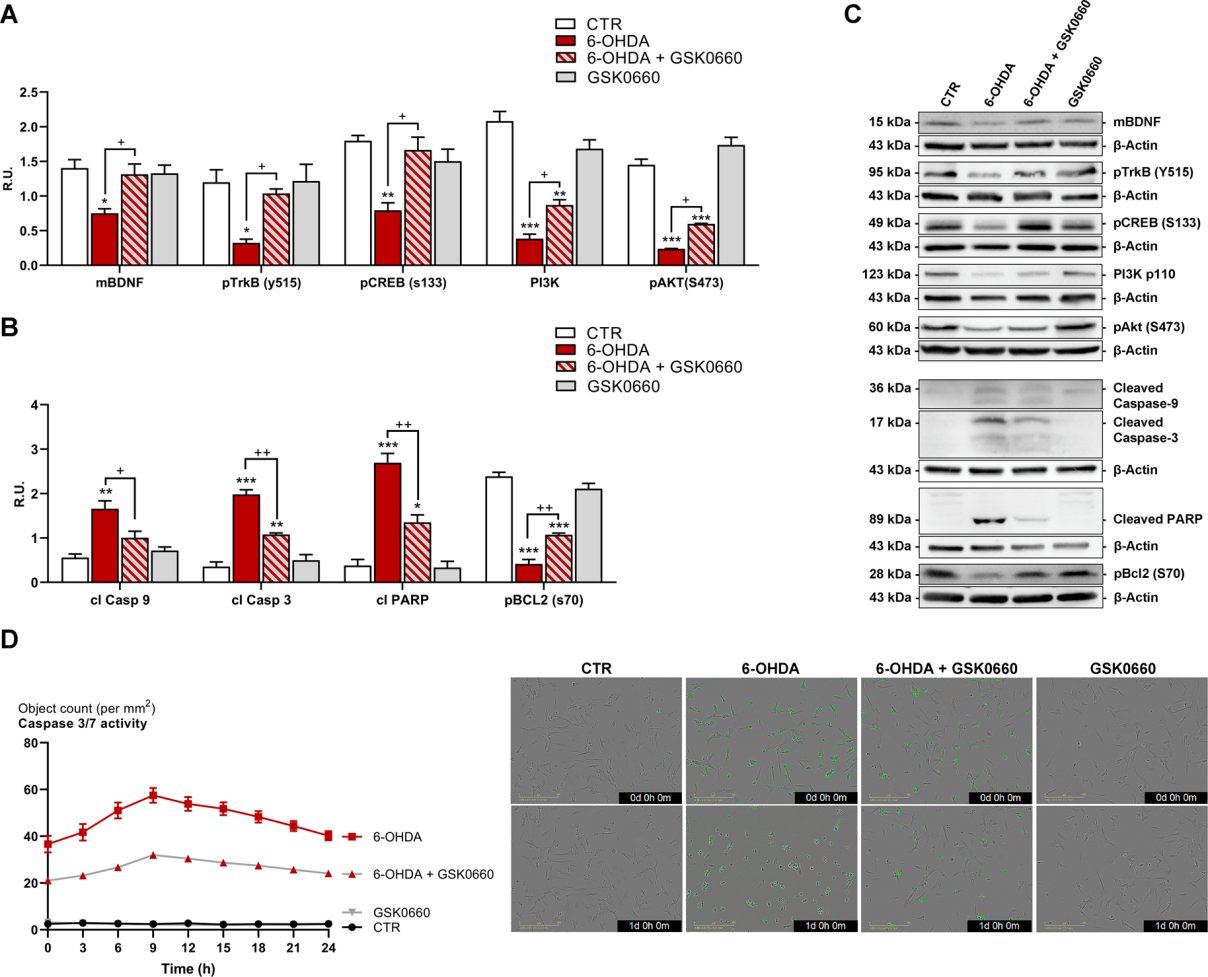
Western blotting representative images (**C**) and relative densitometric analysis for (**S**) mBDNF, pTRKB, pCREB, PI3K, pAKT and (**B**) cleaved Caspase9, cleaved Caspase3, cleaved PARP and pBCL2. (**D**) Live-cell IncuCyte Caspase-3/7 assay for control and treated cells (marked in green). Scale bar: 200 μm

We have already reported a decrease of BDNF/TrkB pathway in neurodegeneration triggered by PPARβ/δ activation [23]; for this reason, proteins involved in survival and death pathways were assayed in our PD in vitro model. Upon 6-OHDA, mature BDNF and its specific receptor TrkB were considerably reduced compared to control cells, while the co-presence of GSK0660 increased these protein levels (Fig. 3A, C). Also, pCREB, controlling mBDNF levels, and the survival pathway PI3K/pAkt were significantly decreased by 6-OHDA and restored at control levels by the antagonist (Fig. 3A, C). These data were further supported by the increase of apoptotic markers (cleaved caspases and PARP), the decrease of pBCL2 and the activation of caspase 3/7 assayed by live-cell imaging upon 6-OHDA challenge, all these parameters were counteracted by the antagonist (Fig. 3B, C and D).

### TaqMan gene expression in the in vitro model

**Fig. 4:**
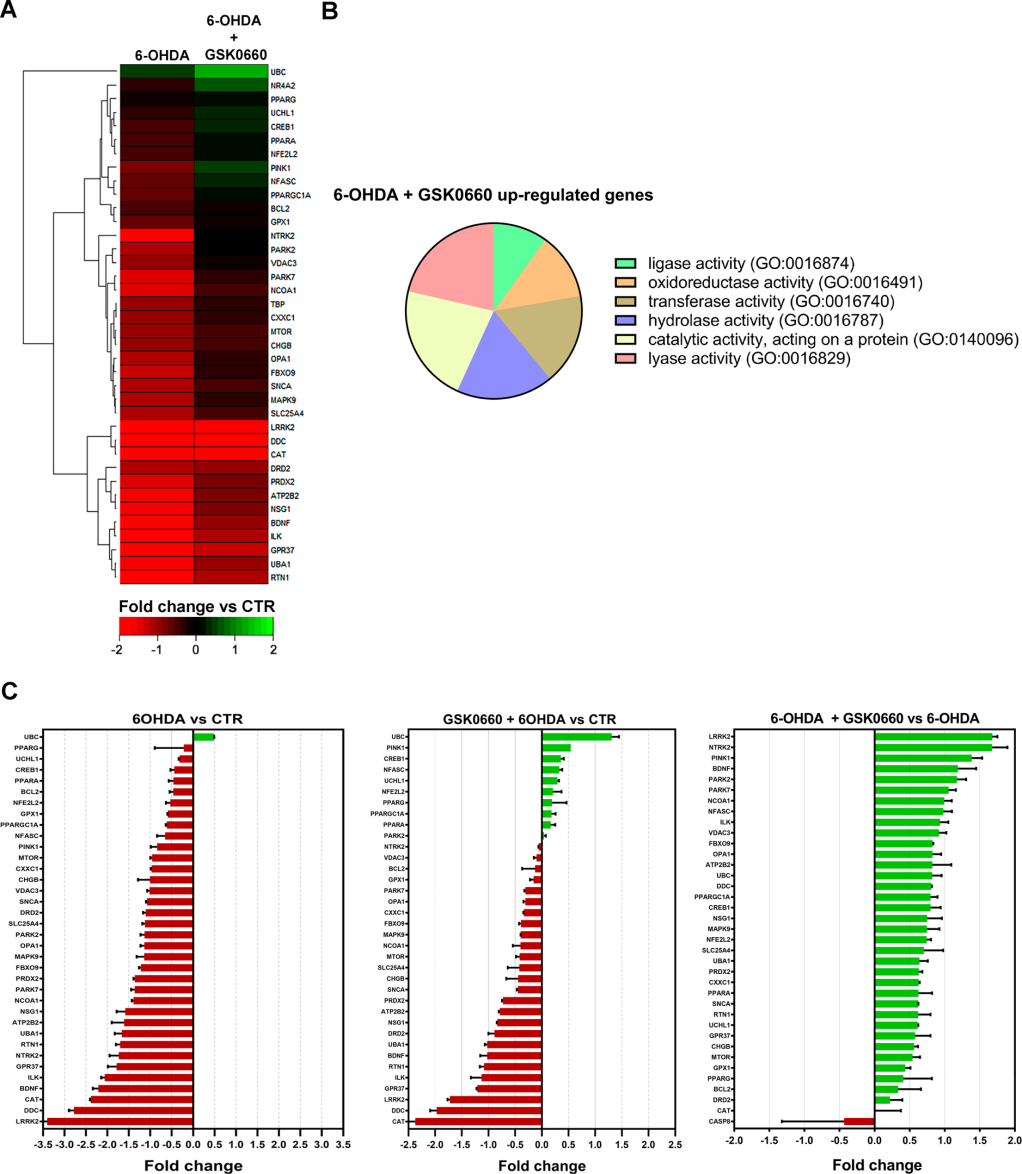
(**A**) Heatmap of overrepresented neuronal function pathways in DEG cluster 3. Heat map showing the clustering of samples by genes that significantly increased (red font) or decreased (green font) in 6-OHDA and 6-OHDA + GSK0660 vs. CTR cells. Red and green colors characterize relatively high and low log2 gene expression values, respectively. (**B**) Pie chart illustration of Panther Gene Ontology biological processes significantly overrepresented in this study. (**C**) Fold change indicates the ratio of the 6-OHDA vs. CTR, 6-OHDA + GSK0660 vs. CTR, 6-OHDA + GSK0660 vs. 6-OHDA groups

Moreover, to evaluate gene expression changes upon different treatments and in particular the effect of GSK0660 in combination with 6-OHDA challenge, TaqMan gene expression array was performed in control and treated cells. Genes assayed in the array were selected since they have a key role in PD, and it has been reported that most of them are reduced in this disease model (6-OHDA challenge) [36]. Indeed, in Table 1, in the heatmap (Fig. 4A) and in the Fig. 4C, it is possible to observe a downregulation of the expression of most of the genes examined in the array upon 6-OHDA, while the antagonist counteracted this effect as you can appreciate from Fig. 4C. Notably, genes modulated are involved in crucial pathways of PD (Table 2), including survival, proteasome activity, ubiquitination, mitochondrial functionality, and neurotrophic support. The results of the array supported the analyses performed so far. Interestingly, the neurotrophic support (BDNF/TrkB/CREB pathway) was upregulated by the presence of the antagonist, confirming its neuroprotective effect. Finally, functional analysis by the Panther software identified gene ontology biological processes, which resulted significantly upregulated by GSK0660 (Fig. 4B).

**Table 2:** List of pathways in which the genes are included. In bold the genes resulting upregulated upon GSK0660 treatment

Parkin complex	PARK7
Parkin substrate	**GPR37**
Cell adhesion	**NFASC**
Ubiquitination	**FBXO9**, **LRRK2, PARK2, PINK1, UBA1, UCHL1**
Inflammation	**PRDX2**
Apoptosis	**MAPK9**, **BDNF**, **OPA1, PRDX2**
Mitochondria	**LRRK2**, **OPA1, PARK7, PINK1, SNCA, UCHL1, VDAC3**
Synaptic vesicles	**LRRK2**
Signal transduction	Dopaminergic: **NSG1, DDC, PARK2, PARK7, PINK1, SNCA** MAP Kinase: **MAPK9, PRDX2**
Ion transport	**ATP2B2**, **CXXC1, SNCA, VDAC3**
Transporters	**VDAC3**
BDNF pathway	**BDNF**, **NTRK2 (TrkB), CREB1**
Others	**NCOA1, RTN1**

### Analyses of mitochondria in the in vitro model

**Fig. 5:**
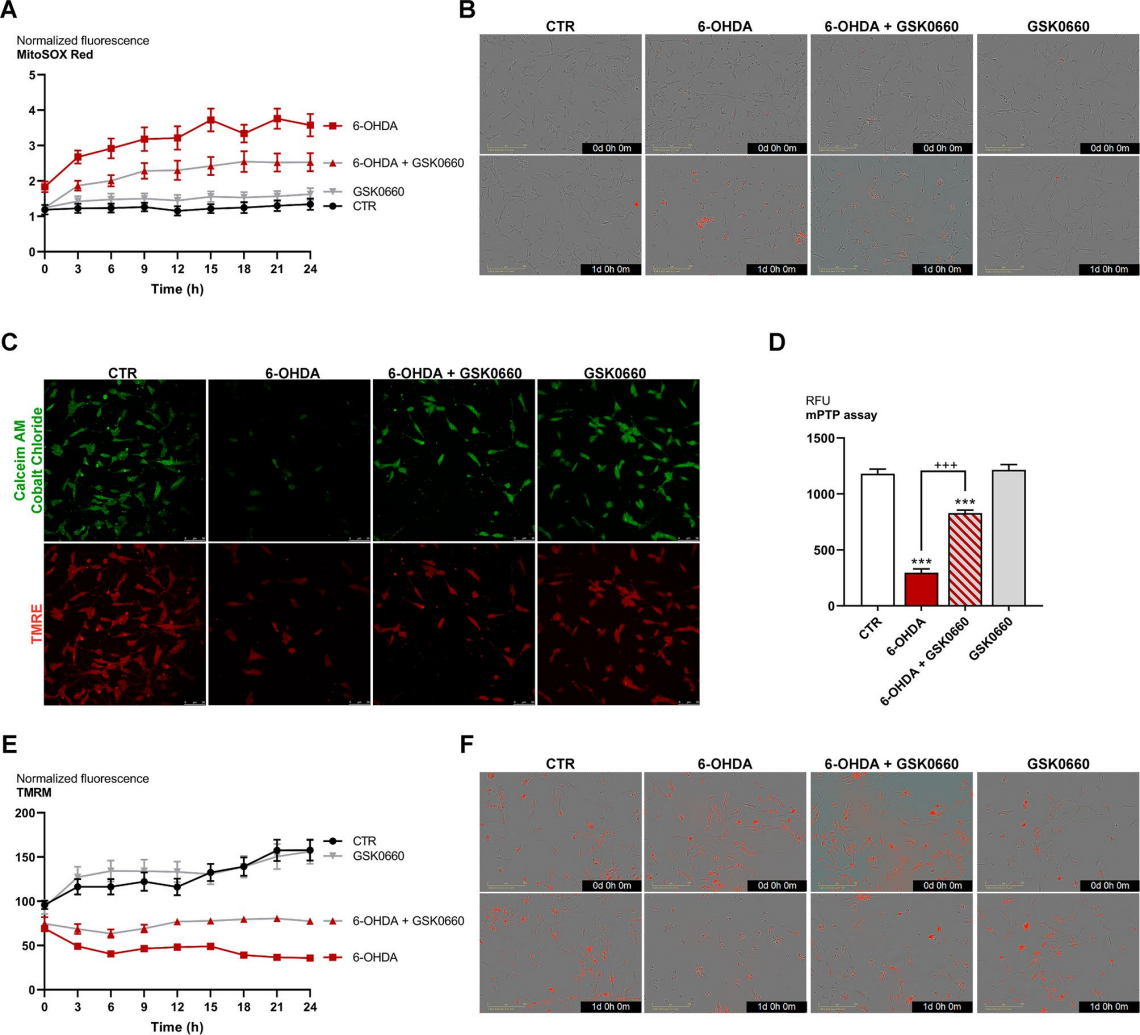
(**A**) Real-time graph and (**B**) representative images of live-cell IncuCyte MitoSox red assay for control and treated cells (marked in red). Scale bar: 200 μm. (**C**) Images of mPTP assay and (**D**) histograms of green fluorescence quantification (calcein-AM/cobalt chloride). Scale bar: 50 μm. (**E**) Real-time graph and (**F**) representative images of live-cell IncuCyte TMRM assay for control and treated cells (marked in red). *** p < 0.0005 vs. control (CTR). ^+++^p < 0.0005 vs. 6-OHDA

Mitochondria dysfunction was analyzed using the MitoSOX fluorescent probe, which reveals the presence of mitochondrial superoxide. Notably, MitoSOX resulted strongly upregulated upon 6-OHDA, while the PPARβ/δ antagonist was able to counteract this effect (Fig. 5A, B). To investigate in-depth, the mitochondrial membrane potential was analyzed by TMRE and mitochondrial pore opening was assessed by confocal microscopy (Fig. 5C, D). Upon 6-OHDA, mitochondrial membrane potential was significantly decreased while mitochondrial pore opening was strongly up-regulated. Notably, the presence of the antagonist restored the control conditions. To further confirm the mitochondrial membrane potential alterations, live-cell imaging of TMRM (tetramethylrhodamine, methyl ester) was performed using IncuCyte device. Interestingly, the co-presence of GSK0660 with 6-OHDA reduced the mitochondrial impairment induced by the neurotoxin (Fig. 5E, F).

**Fig. 6:**
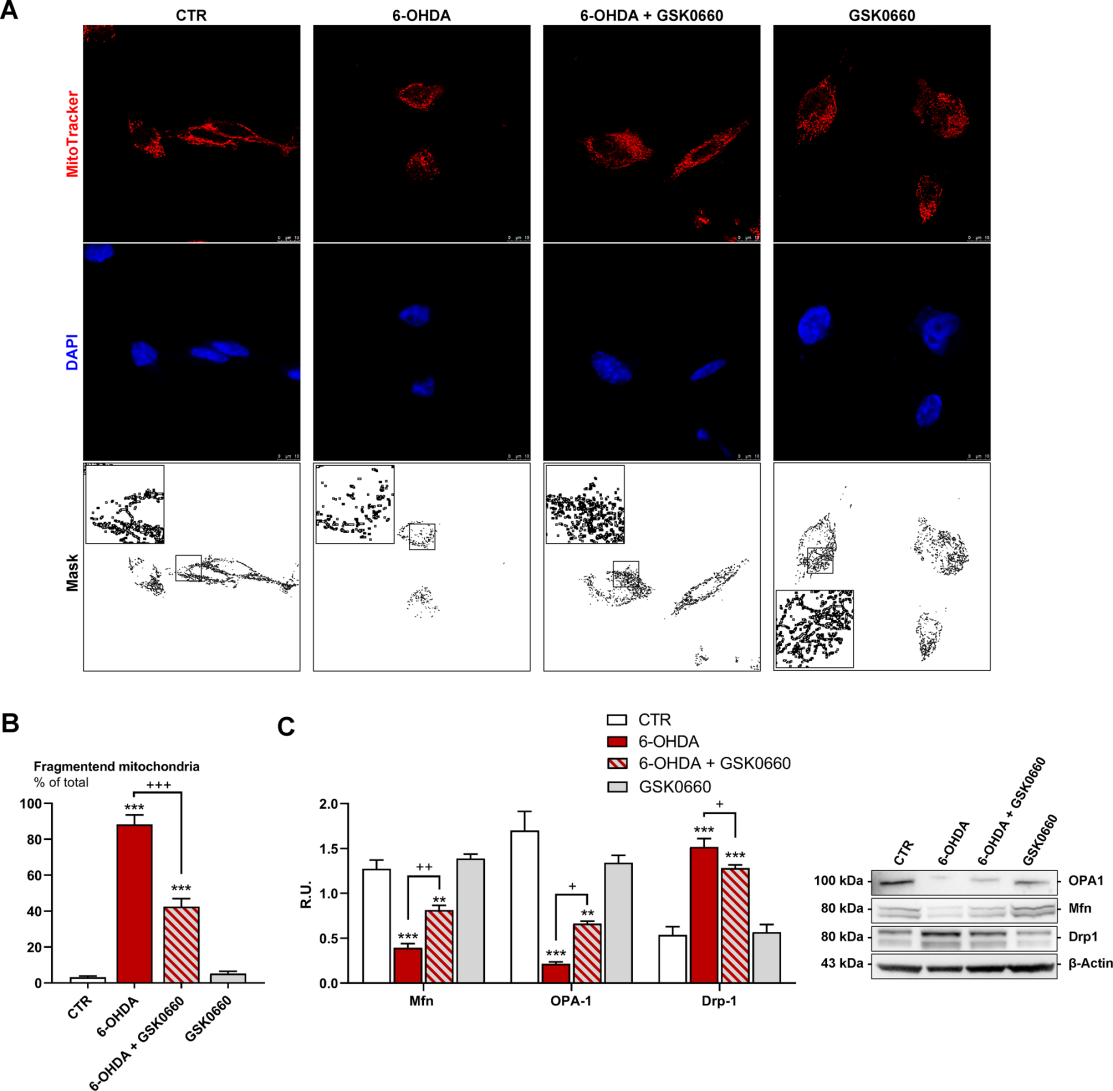
(**A**-**B**) DAPI, Mitotracker deep red, and the masks produced by ImageJ are reported. In the black box, the area is magnified to better appreciate the mitochondrial morphology. (**C**) Western blotting and relative densitometric analysis for fission and fusion pathways. *** p < 0.0005; ** p < 0.005 vs. control (CTR). ^+++^p < 0.0005; ++p < 0.005; +p < 0.05 vs. 6-OHDA (n = 3)

In the light of results obtained on mitochondrial impairment (mitochondrial pore opening, mitochondrial membrane potential alterations and the generation of mitochondrial superoxide), we decided to deeply analyze mitochondrial morphology and mitochondrial fragmentation proteins. Healthy mitochondrial morphology and dynamics are crucial for the maintenance of mitochondrial functionality; thus, to evaluate mitochondrial fragmentation Mitotracker deep Red was used. Mitochondrial morphology of our CTR cells revealed tubular networks, whereas upon 6-OHDA challenge, cells showed shorter and reduced mitochondria, indicating mitochondrial fragmentation. Notably, mitochondrial morphological variations were mitigated by GSK0660 treatment (Fig. 6A, B). Mitochondrial morphology may be due to the interplay between mitochondrial fission and fusion. Mitochondrial fusion is regulated by Mfn1/2 and Opa-1, while mitochondrial fission is mostly controlled by Drp-1. Thus, we analyzed the expression of these factors by Western blot (Fig. 6C). We observed a substantial reduction in Opa-1 and Mfn1/2 upon 6-OHDA challenge, and GSK0660 reduced the decrease in these fusion proteins. The fission protein Drp-1 was increased by 6-OHDA, while the antagonist was able to partially counteract this effect (Fig. 6C).

**Fig. 7:**
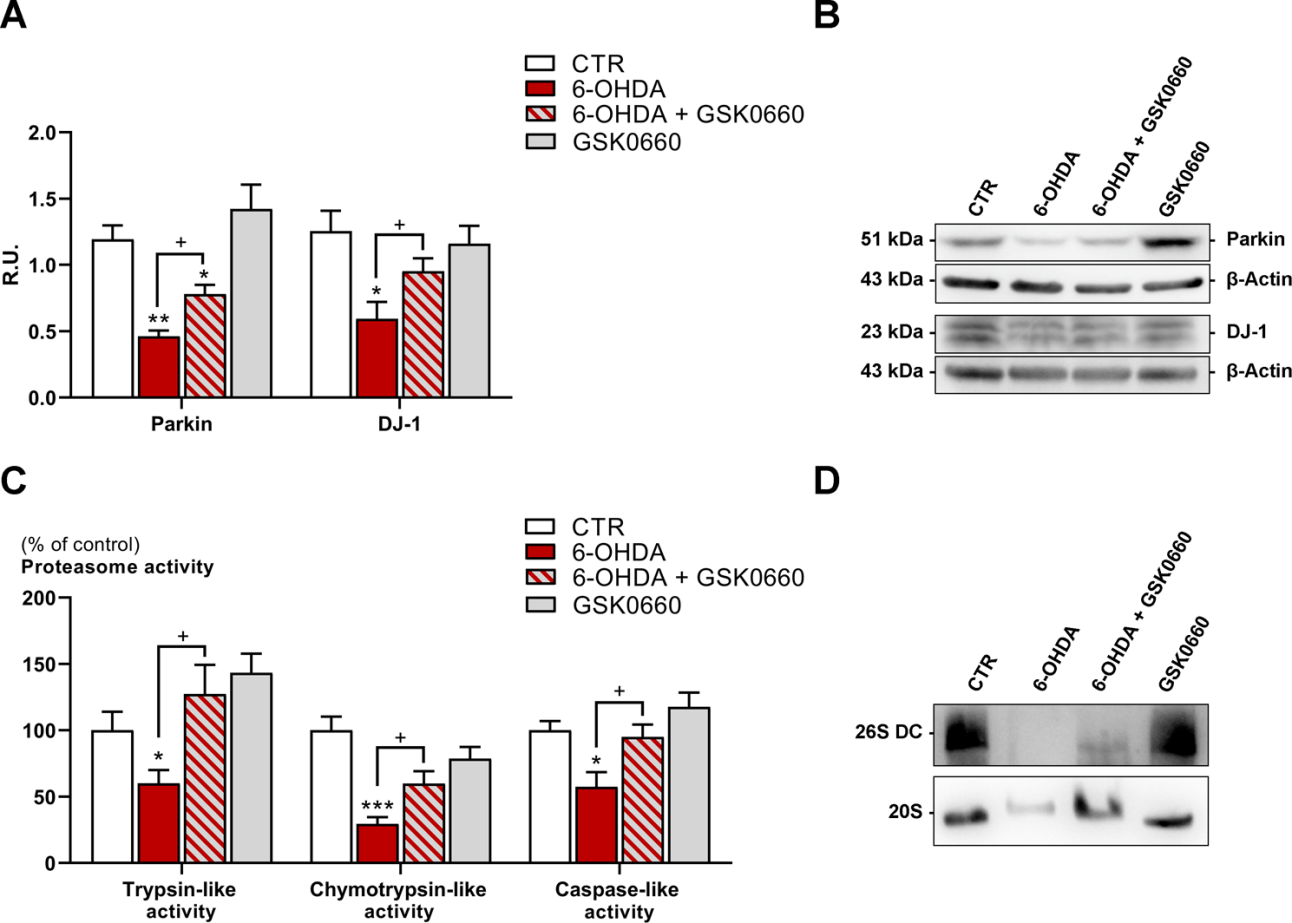
(**A**-**B**) WB and relative densitometric analysis for Parkin and DJ-1 in control and treated cells. (**C**) Proteasome activity assay to evaluate chymotrypsin-like, trypsin-like, and caspase-like activity in control and treated cells. (**D**) Western blotting representative membrane images of proteasome configuration. Data are mean ± SEM of 3 different experiments run in triplicate (n = 3)

To further confirm the mitochondrial dysfunction and the protective effect exerted by the antagonist in our cell model, we investigated the mitochondrial functionality using Mito Stress assay by Seahorse Extracellular Flux Analyzer to study cells’ bioenergetics profile upon treatments (Fig. 7). Specifically, in Fig. 7A time course and live measurement of OCR upon stressor compounds administration is reported. OCR was significantly decreased by 6-OHDA while the co-presence of GSK0660 counteracted this effect (Fig. 7A). Upon oligomycin, cells treated with 6-OHDA and GSK0660 maintained much higher OCR than 6-OHDA alone, parallel with substantial variations in ATP production through mitochondrial respiration and coupling efficiency (Fig. 7B). Induction of maximal respiration with FCCP injection led reduced oxygen consumption upon 6-OHDA, whereas the combined treatment revealed a behavior comparable to CTR condition. Proton leak not resulted significantly varied upon treatments. In addition, 6-OHDA-treated cells showed lower non-mitochondrial respiration, while the antagonist counteracted this effect. Upon rotenone/antimycin A, 6-OHDA cells revealed an extremely low spare respiratory capacity, and also in this case, the presence of the antagonist significantly counteracts this effect (Fig. 7B).

Taken together these results indicated that GSK0660 was able to rescue mitochondria, exerting strong positive effects on mitochondria metabolism and oxidative stress induced by 6-OHDA.

### Analyses of proteasome in the in vitro model

**Fig. 8:**
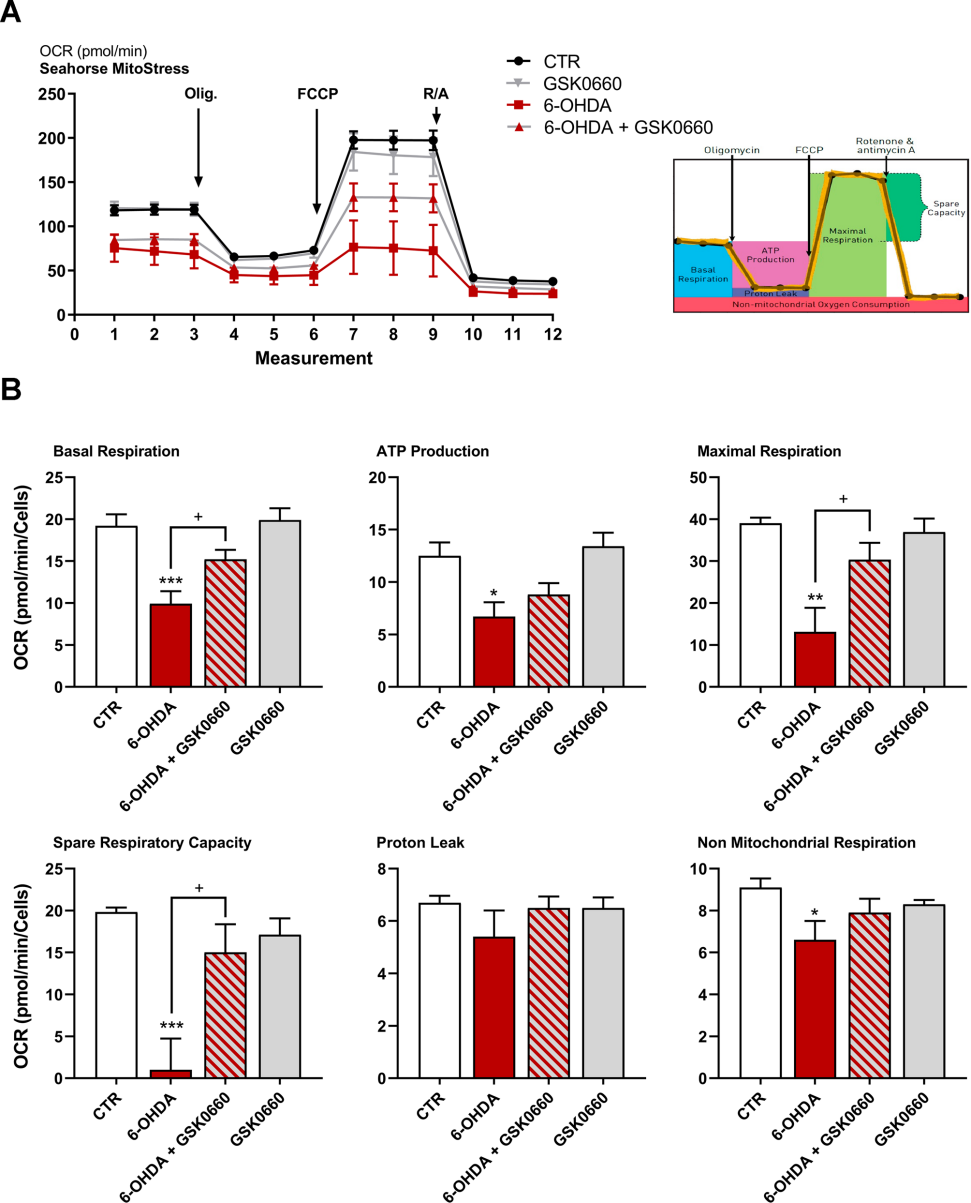
(**A**) Seahorse XF Cell MitoStress Test profile with the key parameters of mitochondrial function namely basal respiration, ATP production, proton leak, maximal respiration, and spare respiratory capacity. (**B**) Statistical analysis of the individual MitoStress test parameters. *** p < 0.0005; ** p < 0.005; * p < 0.05 vs. CTR. ^+^p < 0.05 vs. 6-OHDA

Subsequently, proteasome activity and functionality were assayed by Western blotting analysis specifically for Parkin, DJ1 and proteasome subunits and by fluorescence assay, for chymotrypsin, trypsin and caspase-like activities (Fig. 8). We first evaluated the levels of two proteins essential for ubiquitin-proteasome system (UPS) activity, usually involved in the pathogenesis of sporadic and genetic PD, such as Parkin (PARK2) and DJ-1 (PARK7). Parkin is an E3-ubiquitin ligase, which plays a crucial role in recovering and degrading damaged proteins [37, 38]. Some of its substrates for ubiquitination are α-synuclein and itself [37]. DJ-1 is a small protein ubiquitously expressed; it acts as an oxidative stressor sensor and it is involved in different molecular mechanisms related to oxidative damage dependent-cell response [39–41]. In this regard, DJ-1 plays a crucial role in regulating proteasome activity and maintaining mitochondrial homeostasis [16, 42]. 6-OHDA exposition negatively modulates these proteins’ levels compared to untreated cells (Fig. 8A, B). Conversely, the co-treatment with GSK0660 restored the Parkin and DJ-1 protein levels toward the levels of untreated cells. 26 S proteasome activity was assessed by a specific fluorescence assay, as previously described [27]. Each activity of proteasome (chymotrypsin-, trypsin- and caspase-like) was evaluated, and it is possible to observe that 6-OHDA alone induced a significant decrease of each proteasome activity, reflecting the behavior of proteasome in PD dopaminergic neurons. The 6-OHDA effect on 26 S proteasome activity was completely reverted for trypsin-like activity and partially reverted for chymotrypsin- and caspase-like activities in the presence of GSK0660 (Fig. 8C). Moreover, the proteasome characterization by Western blotting revealed a strong downregulation of the proteasome subunits 26 S and 20 S by 6-OHDA, while the presence of GSK0660 slightly counteract this effect (Fig. 8D).

### Silencing of PPARβ/δ in the in vitro model

Finally, to validate the hypothesis regarding the involvement of PPARβ/δ activation in the neurotoxicity, PPARβ/δ siRNA was performed, and the silenced cells were analyzed by Western blotting and viability assay (Suppl Fig. 1A, B). A significant reduction of PPARβ/δ in silenced cells than scramble is observed, confirming that gene silencing has occurred (Suppl Fig. 1A). In the same supplementary Fig. 1B, the MTS assay, showing neuronal rescue in PPARβ/δ silencing-6-OHDA treated cells, confirmed this protein’s involvement in the neurotoxicity, highlighting the effect of PPARβ/δ silencing in reducing the 6-OHDA-neurotoxic impact. Notably, the Western blotting for pCREB protein and real time PCR for CREB and BDNF genes confirmed the role of PPARβ/δ in 6-OHDA-induced injury, showing increased phosphorylation of CREB in silencing conditions upon 6-OHDA, paralleled with an upregulation in BDNF and CREB expression, reflecting previous results obtained using PPARβ/δ antagonist (Suppl. Figure 1 C, D).

### Effects of GSK0660 in a hemi-lesioned PD mouse model

**Fig. 9:**
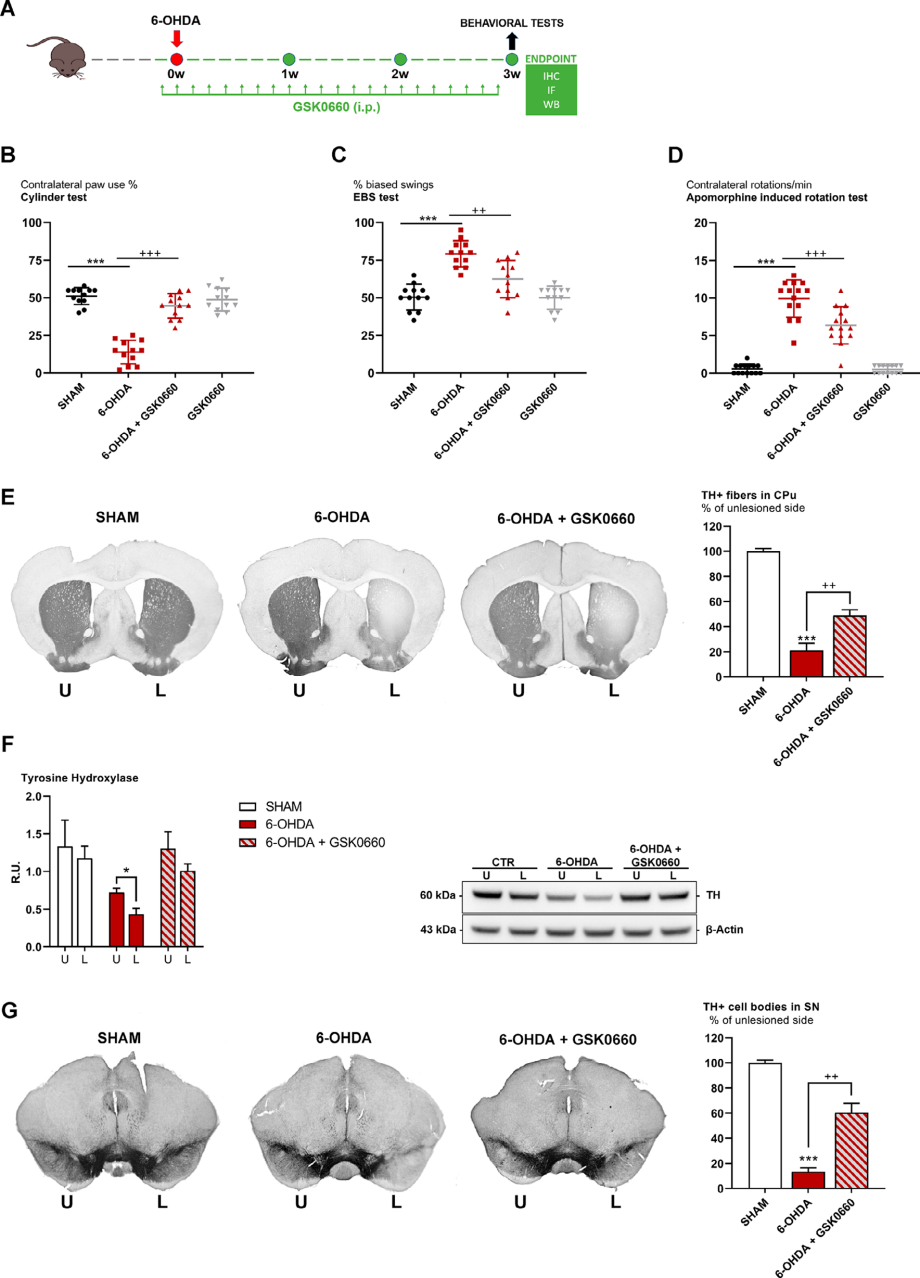
(**A**) Experimental design. (**B**) Cylinder test, (**C**) EBS test and (**D**) Apomorphine induced rotation test in SHAM, 6-OHDA, 6-OHDA + GSK0660 AND GSK0660 animals. Transverse section taken through the striatum (**E**), immunostained for TH to evaluate the dopaminergic-induced injury by stereotaxic injection of 6-OHDA in the right side. Histograms shows the percentage of TH + fibers loss in striatum (CPu) (expressed in percentage of unlesioned side). (**F**) Western blotting and relative densitometric analysis for TH in the striatum. Transverse section taken through substantia nigra pars compacta (**G**), immunostained for TH to evaluate the dopaminergic-induced injury by stereotaxic injection of 6-OHDA in the right side. Histogram shows the TH + cell bodies in substantia nigra (SN) (expressed in percentage of unlesioned side). *** p < 0.0005, vs. CTR; ^+++^ p < 0.0005, ^++^ p < 0.005 vs. 6-OHDA

In light of the encouraging data obtained in the in vitro model, GSK0660 was tested in a hemi-lesioned 6-OHDA mouse model. This PD in vivo model was validated by behavioral tests as described in our previous work [30]. The apomorphine and motor tests, such as cylinder and EBST tests, were performed in hemi-lesioned animals treated or not with GSK0660 (Fig. 9A-D). The presence of GSK did not affect body weight (**data not shown**), while rescued animal behavior and apomorphine-induced rotation test (Fig. 9A-D).

For assessing the potential protective effects of the antagonist in counteracting 6-OHDA-inducing alteration of dopaminergic neurons, we analyzed TH immunoreactivity. TH is the rate-limiting enzyme responsible for the biosynthesis of dopamine [43]. TH-immunoreactivity is thus a measure of the functionality of dopaminergic neuronal cells. In Fig. 9E and G, the immunostaining of TH in dopaminergic neurons was reported both in *striatum* and *substantia nigra* (Fig. 9E, G, respectively). In 6-OHDA-treated mice, a strong reduction of TH immunoreactivity was observed, whereas the antagonist rescued dopaminergic neurons in both *substantia nigra* and *striatum*, confirming a neuroprotective effect. (Fig. 9E, G). These results were also confirmed by Western Blotting of *striatum* (Fig. 9F).

**Fig. 10:**
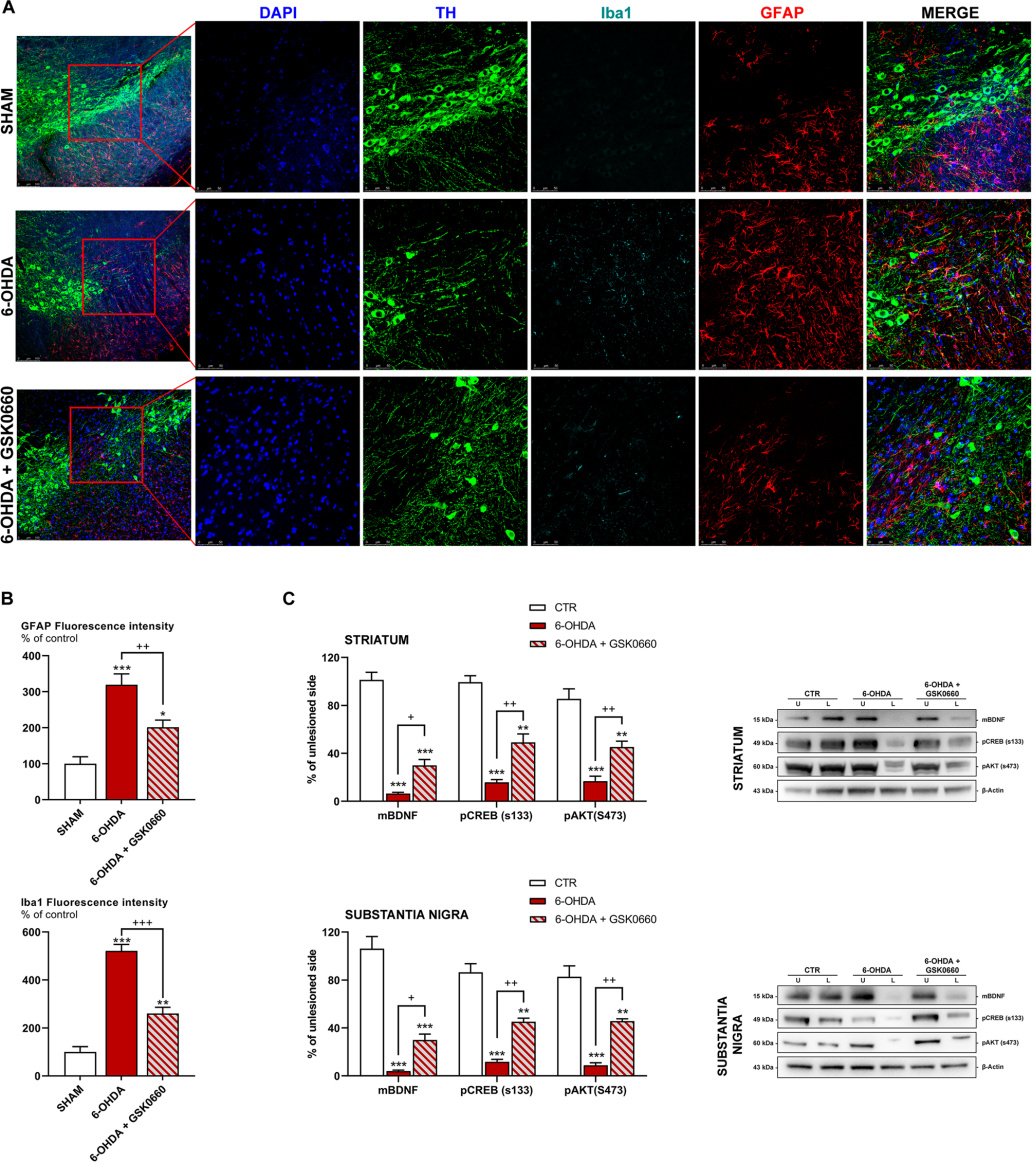
(**A**) Triple immunostaining at 40x magnification for Iba1, TH and GFAP, nuclei were counterstained with DAPI. On the left, it is possible to appreciate 20x magnification, while on the right higher magnification for TH, Iba1 and GFAP staining and merge figures were shown. (**B**) Histograms for GFAP and Iba1 show the fluorescence intensity, as % of controls. (**C**) Western blotting and relative densitometric analysis for mBDNF, pCREB and pAKT in the *striatum* and *substantia nigra* *** p < 0.0005; ** p < 0.005; * p < 0.05 vs. CTR; ^+++^ p < 0.0005, ^++^ p < 0.005 vs. 6-OHDA.

To investigate the effects of the antagonist on the neuroinflammatory processes and in the protection of dopaminergic neurons, triple immunofluorescence analyses for the inflammatory markers Iba1, GFAP (markers of activated microglia and astrocytes, respectively), and TH in *substantia nigra* coronal brain sections of the same animals are reported (Fig. 10A). It is possible to appreciate a sharp increase in intensity of GFAP and Iba1 in 6-OHDA brain sections, whereas GSK0660 was able to prevent 6-OHDA-induced effects, thus suggesting a decrease in neuroinflammation and gliosis and a protection of dopaminergic neurons (Fig. 10A, B). To further support the positive effects on dopaminergic neuronal loss, we assayed the neurotrophic and pro-survival proteins mBDNF, pCREB and pAKT in *striatum* and *substantia nigra* by Western Blotting. Interestingly, in line with in vitro studies, the antagonist counteracted the detrimental effects of 6-OHDA, significantly increasing the level of these proteins, thus confirming the neuroprotective effects exerted by GSK0660 (Fig. 9C).

## Discussion

The underlying mechanisms of PD are still unclear but oxidative stress, neuroinflammation and decreased neurotrophic support are involved. The PD in vitro models, such as those obtained by treatment with 6-OHDA, which destroys dopaminergic neurons by generating reactive oxygen species [44], are well-recognized models of the disease. Besides the pro-oxidant effects of 6-OHDA, it has also been described that this compound can lead, through the neuronal development period, architectural alterations in the *substantia nigra* due to BDNF lack [45]. BDNF, a neurotrophin family member, has a key role in the survival, proliferation, and differentiation of neural cells [8]. Neurotrophins have been identified as crucial regulators of plasticity and synaptic processes and attracted great interest concerning neurodegenerative diseases including PD [46–48]. Numerous neurotrophins in the brain have been identified as support for adult neurons’ survival and neuroprotective agents in PD models [49]. BDNF, Nerve growth factor (NGF), and GDNF prevented neuronal degeneration of the lesioned nigrostriatal system in vivo [6, 50] and improved motor impairment [51]. These changes occurring upon 6-OHDA challenge suggest that neurotrophins imbalance triggers other downstream molecules involved in PD pathogenesis. It has been demonstrated that BDNF therapy counteracts neurodegenerative processes and supports brain repair [52, 53]. Notably, antioxidants increase dopamine production in the brain, which in turn stimulates BDNF synthesis and release, thus stimulating neurons’ growth via the TrkB pathway [54, 55]. Also, the endogenous production of BDNF is responsible for preventing neurodegeneration in *substantia nigra* tissue [56].

In this regard, we have previously demonstrated that, during aging, PPARβ/δ plays a detrimental role in different neurodegenerative disorders by decreasing mBDNF and TRKFl and inducing death pathways depending on p75 activity [23]. In agreement, in the present study, we demonstrate that 6-OHDA induces, likely by PPARβ/δ activation, a dramatic decrease of cell viability paralleled by an overall downregulation of the survival pathway BDNF/TrKBFl, while causing the death pathways triggered by p75. These alterations occurred after treatment with 6-OHDA, indicating that neurotrophic factors and oxidative stress imbalance are triggers for the activation of downstream signals involved in PD’s pathogenesis. In support of this concept, PPARβ/δ antagonist treatment acted in the recovery of BDNF after 6-OHDA treatment in vitro. This event may lead to dopaminergic neurons’ rescue since, loss of dopaminergic neurons is the main characteristic of this disease [57].

We have previously demonstrated that 6-OHDA hemi-lesioned animals showed an increase of oxidative stress, highlighted by 4-HNE protein adducts [23]. HNE has been indicated as a PPARβ/δ ligand and contributed to its activation, a known oxidative stress sensor, which induces TrkBfl reduction and therefore cell death [23]. Furthermore, a relationship between pro-BDNF signaling and PPARβ/δ was reported in Alzheimer’s disease in vitro and in vivo models [12].

It has been postulated that one of primary mechanism of dopaminergic neuronal loss is oxidative stress due to 6-OHDA challenge [58]. In fact, upon toxin treatment, differentiated SH-SY5Y increased HNE levels, which likely act as PPARβ/δ ligand, while the antagonist restored HNE control values. PPARβ/δ antagonist treatment protected the dopaminergic neurons from 6-OHDA challenge, and this protection may be related to a decrease of oxidative stress as also observed for the levels of oxidized proteins, restored to control value by the antagonist.

The neuroprotective action of PPARβ/δ antagonist in the PD cellular model may be due to increased neuronal survival that requires neurotrophic support, mainly BDNF but may be attributable also to a general decrease of oxidative stress by the antagonist, since it is already reported that high levels of oxidative stress are responsible for a decrease of the neurotrophic support. These findings align with the gene expression reported in Tables 1 and 2, where a consistent decrease of BDNF and TrkB gene expression was observed upon 6-OHDA, prevented by the antagonist’s presence. In the same manner, antioxidant genes are downregulated by the neurotoxin and restored by the antagonist. Finally, genes involved in proteasome and mitochondrial functionality appear all negatively modulated by 6-OHDA. Overall, crucial genes involved in PD resulted downregulated by 6-OHDA challenge, while the co-treatment was able to revert this behavior, thus confirming the neuroprotective effects exerted by the antagonist GSK0660.

This study revealed a crucial role of GSK0660 in mitochondrial protection. Upon 6-OHDA, a mitochondrial dysfunction (as reported by Seahorse analysis, fragmentation, TMRM and mPTP assays), possibly correlated to the oxidative condition, is evident concurring together with neurotrophic factors reduction to dopaminergic neuronal death. All these effects were counteracted by the antagonist inducing neuronal survival. In particular, GSK0660 was able to reduce the mitochondrial pore opening, mitochondrial membrane potential alterations, the generation of mitochondrial superoxide and the fragmentation, parallel with a rescue of mitochondrial metabolism (all parameters altered by 6-OHDA challenge).

We suggest also that the inhibition of proteasome activity observed may be due to the formation of 4-HNE protein adducts with specific catalytic subunits into proteasome hole, as already demonstrated [59, 60]. Moreover, in this scenario, the site deputed to chymotrypsin-like activity is more susceptible to 4-HNE damage, as previously reported [59]. Thus, considering that GSK0660 exposition counteracts the higher amount of 4-HNE protein adducts induced by 6-OHDA, we hypothesize that the antagonism of PPAR β/δ may restore the essential molecular processes leading to the turnover of damaged proteins, such as UPS and proteasome activity. On the other hand, it is known that an increase of oxidized proteins, as observed by Oxyblot analysis, may determine an overload of oxidized proteins in the proteasome leading to its dysfunction.

In summary, our findings suggest that PPARβ/δ antagonist has neuroprotective potential due to neurotrophic support, anti-apoptotic activity, improved mitochondrial respiration, mitochondrial dynamic and proteasome activity amelioration. We show that the antagonist not only decreases oxidative stress and oxidized proteins, contributing to proteasome functionality, but it is also able to activate Akt, and CREB thus leading to BDNF and TRkBFl increase. Besides, mitochondrial membrane potential resulted restored, while mitochondrial impairment and oxidative stress was reduced at control levels. These findings are strongly supported also by the siRNA results demonstrating that by silencing PPARβ/δ a significative rescue of the dopaminergic neurons was obtained, thus indicating an involvement of PPARβ/δ in PD’s pathogenesis. Moreover, since PPARβ/δ antagonism affects all the mentioned pathways analyzed, the present investigation strengthens our previous findings on PPARβ/δ as a therapeutic target for neurodegenerative disorders.

Interestingly, in the in vivo model GSK0660 treatment confirmed the neuroprotective effect observed in the in vitro studies. Indeed, the antagonist led a substantial decrease of dopaminergic neuronal loss induced by 6-OHDA (as highlighted by TH immunochemistry). This neuroprotective effect was confirmed by the behavioural performance and apomorphine rotation tests amelioration. In addition, imaging and western blotting strengthen our evidence regarding the tested compound, since we observed a decrease of astrogliosis and activated microglia, concomitant with an upregulation of mBDNF, pCREB and pAKT.

## Conclusion

In concert, PPARβ/δ antagonist displayed neuroprotective activities against the 6-OHDA-induced PD models both in vitro and in vivo, suggesting that it may represent a novel therapeutic approach for PD. These neuroprotective effects are postulated to be due to an antioxidant potential and inhibitory role in various critical events implicated in neurodegeneration, preserving neurotrophins levels, and regulating mitochondrial proteasome functions, which consequently rescue dopaminergic neurons.

The present observations of GSK0660-mediated rescue of the detrimental effects of 6-OHDA challenge were highlighted in vitro, and in vivo, however, further investigations are necessary to deeply dissect the underlying effect of the protective activity of the antagonist. Additional investigations of testing GSK0660 in other in vitro models such as induced pluripotent stem cells or primary neural cells, could be interesting. Finally, the in vivo studies were performed administering the antagonist prior 6-OHDA inoculation, thus future studies can be focused on evaluating the effect of the antagonist post-injury or using another PD animal model such as MPTP or α-synuclein, which begs the question of whether the neuroprotective mechanisms of GSK0660’s rescue of dopaminergic neurons endures from acute to chronic phases of PD. Despite these limits, the current results propose novel pathways of neuroprotection on which to build upon strategies to abolish dopaminergic neuronal loss and suggest that GSK0660 may represent a novel therapeutic approach for PD.

## Electronic supplementary material

Below is the link to the electronic supplementary material.

Supplementary Material 1

Supplementary Material 2

## Data Availability

The data that support the findings of this study are available from the corresponding author upon reasonable request.

## Abbreviations

PD: Parkinson’s disease
PPARs: Proliferator activated receptors
mBDNF: between mature brain-derived neurotrophic factor
pro-BDNF: immature form brain-derived neurotrophic factor
TrkB: Tropomyosin receptor kinase B
TrkBFl: full-length TrkB
4-HNE: 4-Hydroxynonenal
6-OHDA: 6-hydroxydopamine
DMEM: Dulbecco’s minimum essential medium
FBS: fetal bovine serum
ATRA: All-Trans Retinoic acid
TPA: 12-O-tetrade-canoyl-phorbol-13-acetate
CI: Cell Index
BCA: Bicinchoninic acid
PVDF: polyvinylidene difluoride
TH: Tyrosine hydroxylase
ROS: Reactive oxygen species
TBS-T: tris-buffered saline
DCFDA: 2′–7′-dichlorofluorescein diacetate
PBS: Phosphate Buffered Saline
SOD: Superoxide dismutase
BSA: Bovine serum albumin
DAPI: 4′,6-diamidino-2-phenylindole
HBSS: Hanks’ Balanced Salt Solution
DTT: dithiothreitol
MMP: Mitochondrial Membrane Potential
TMRE: Tetramethylrhodamine, ethyl ester
siRNA: Small interference RNA
EBST: elevated body swing test
NGF: Nerve growth factor
GDNF: Glial cell line-derived neurotrophic factor
UBS: ubiquitin-proteasome system
