# Supplementary material for: Neuroprotective effects of the PPARβ/δ antagonist GSK0660 in in vitro and in vivo Parkinson’s disease models
Source: Biol Res. 2023 May 25;56:27. doi: 10.1186/s40659-023-00438-1 (PMC10210307; doi:10.1186/s40659-023-00438-1)

Uncropped WB

Legend:
1 = CTR; 2 = 6-OHDA; 3 = 6-OHDA + GSK0660; 4 = GSK0660.

Figure 2

Cytoplasmatic PPARβ/δ

1 – 2 – 3 – 4

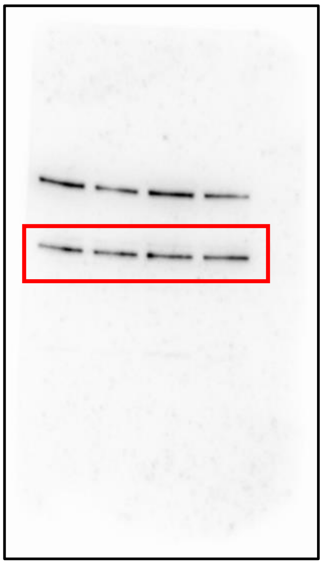

Cytoplasmatic PPARβ/δ

1 – 2 – 3 – 4

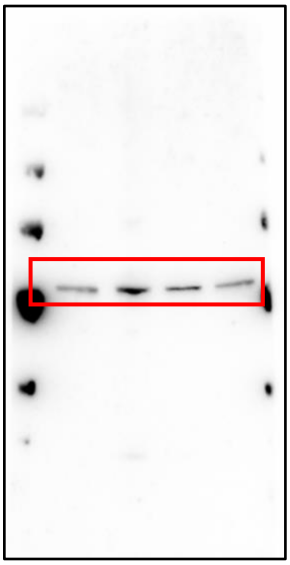

Cytoplasmatic GAPDH

1 – 2 – 3 – 4

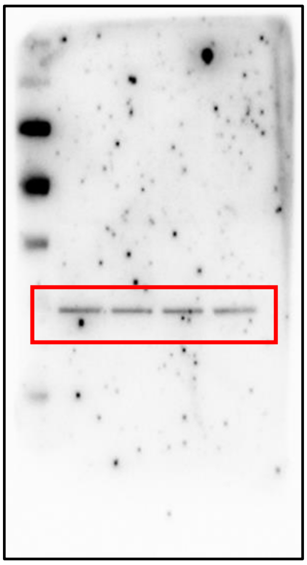

Cytoplasmatic GAPDH

1 – 2 – 3 – 4

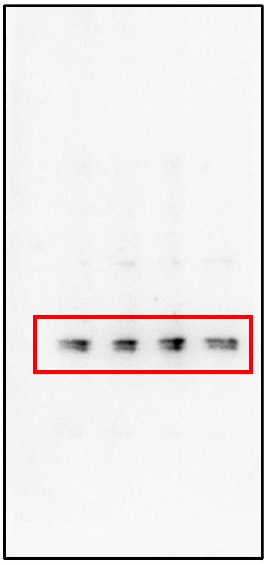

Figure 3

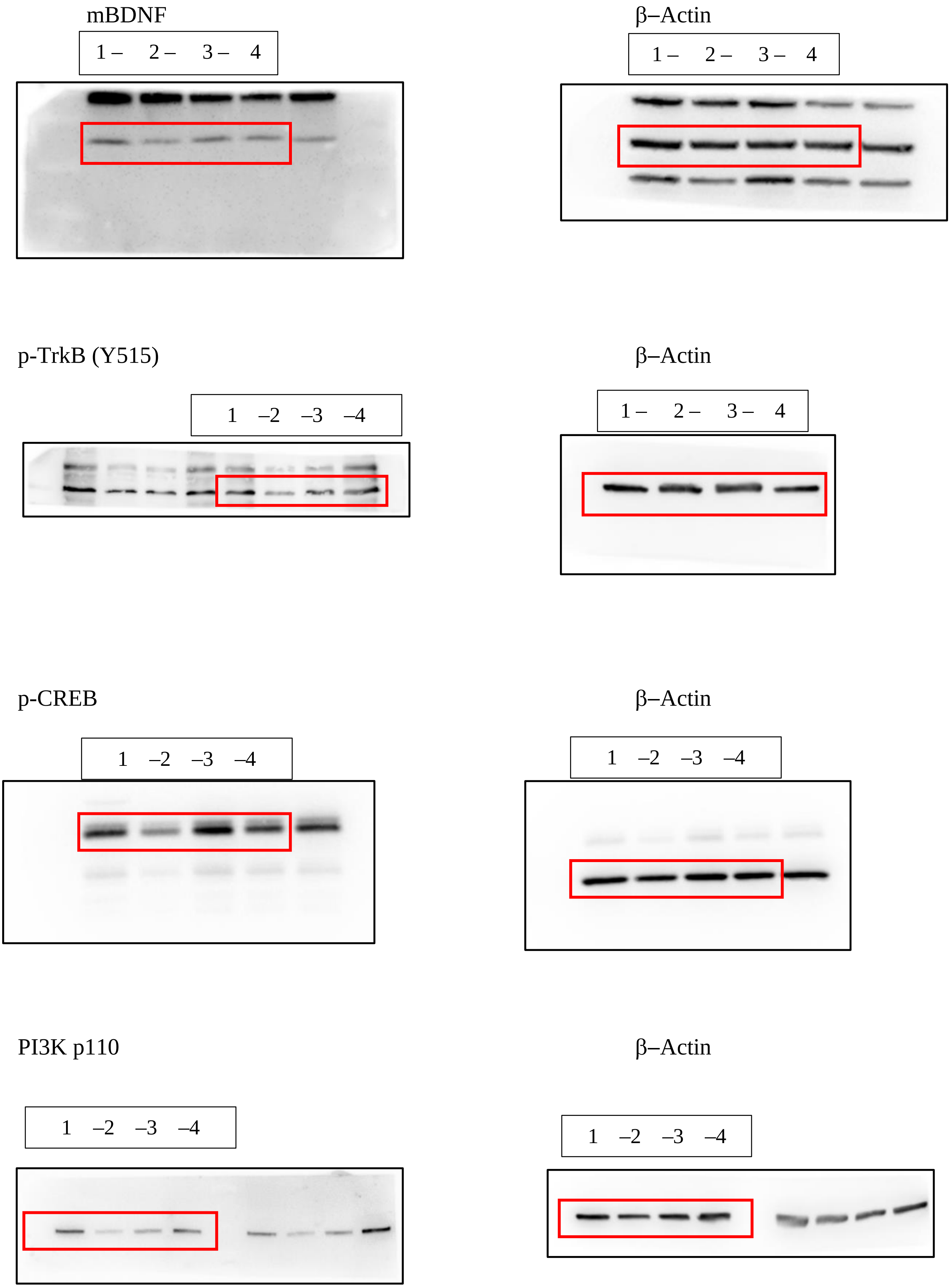
mBDNF
1 – 2 – 3 – 4
β–Actin
1 – 2 – 3 – 4
p-TrkB (Y515)
1 –2 –3 –4
β–Actin
1 – 2 – 3 – 4
p-CREB
1 –2 –3 –4
β–Actin
1 –2 –3 –4
PI3K p110
1 –2 –3 –4
β–Actin
1 –2 –3 –4

p-AKT

1 –2 –3 –4

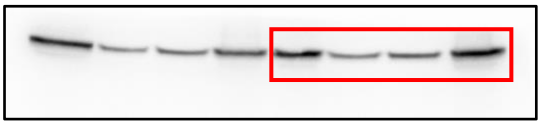

β–Actin

1 –2 –3 –4

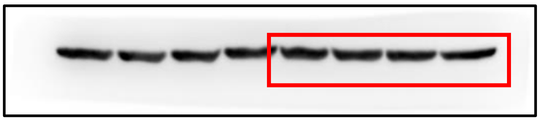

Cleaved Caspase 9

1 –2 –3 –4

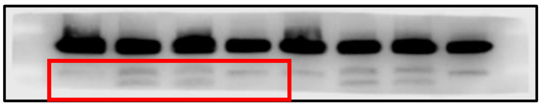

Cleaved Caspase 3

1 –2 –3 –4

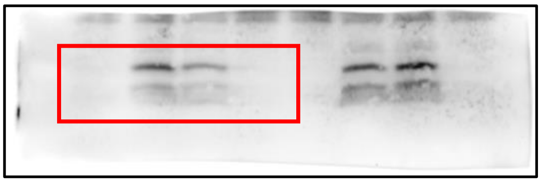

β–Actin

1 –2 –3 –4

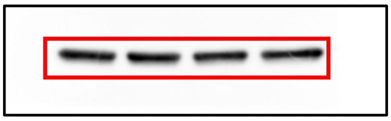

p-Bcl2 (S70)

1 –2 –3 –4

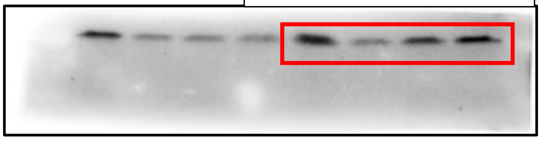

β–Actin

1–2–3–4

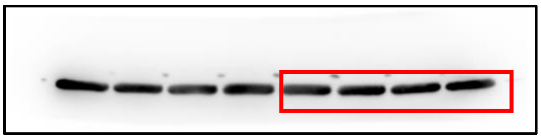

Cleaved PARP

1 –2 –3 – 4

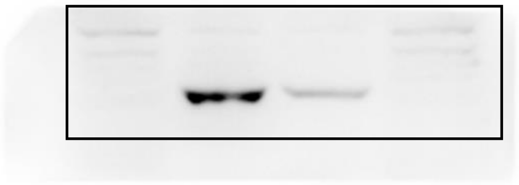

β–Actin

1 –2 –3 – 4

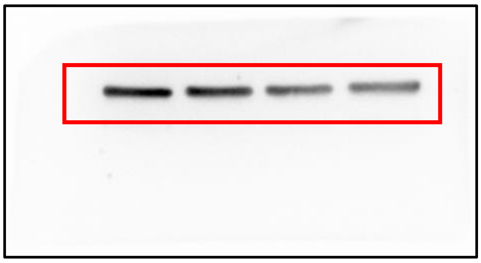

Figure 6

Opa-1

1 –2 –3 –4

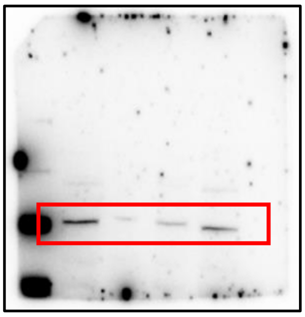

Mfn

1 –2 –3 –4

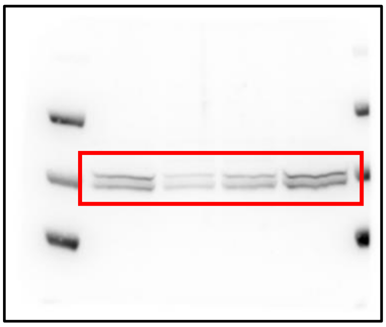

DRP-1

1 –2 –3 –4

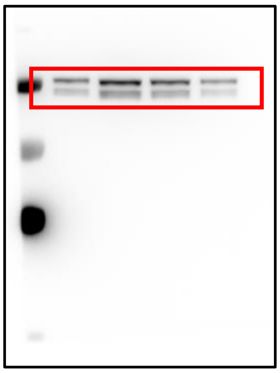

β–Actin

1 –2 –3 –4

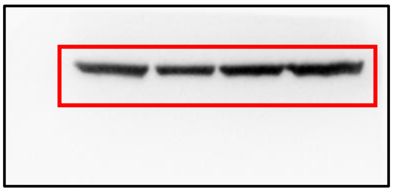

Figure 8

Parkin

1 –2 –3 –4

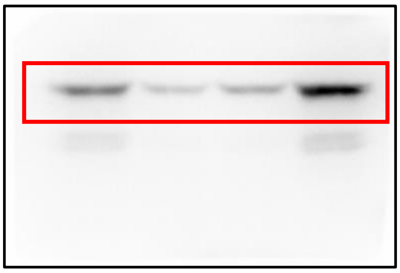

β–Actin

1 –2 –3 –4

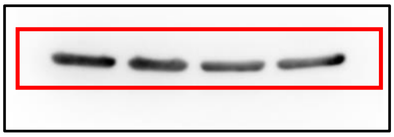

Dj1

1 –2 –3 –4

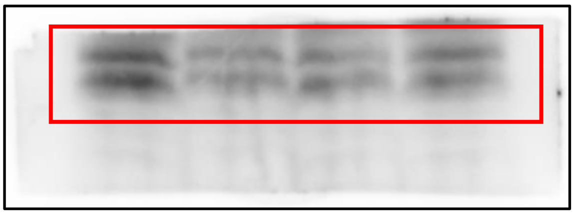

β–Actin

1 –2 –3 –4

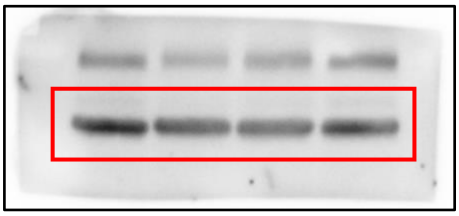

Figure 10

Legend:
1 = CTR Unlesionate; 2 = CTR Lesionate; 3 = 6-OHDA Unlesionate; 4 = . 6-OHDA Lesionate; 5 = 6-OHDA + GSK0660;

mBNDF (Striatum)

1 – 2 – 3 – 4 – 5 – 6

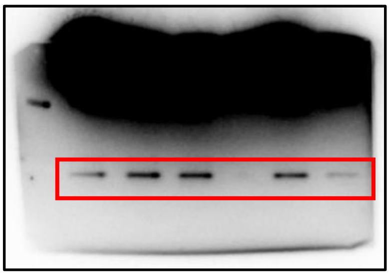

p-AKT (Striatum)

1 – 2 – 3 – 4 – 5 – 6

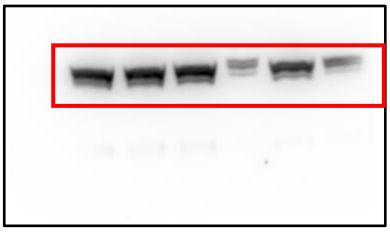

β–Actin (Striatum)

1 – 2 – 3 – 4 – 5 – 6

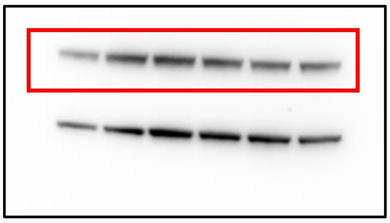

mBDNF (Substantia Nigra)

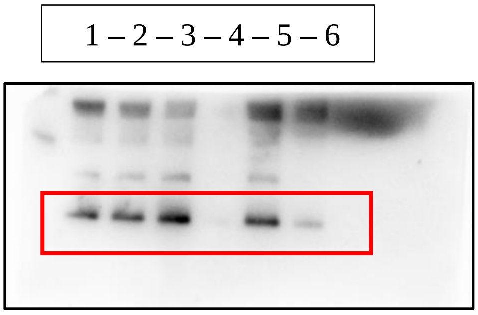

pCREB (Substantia Nigra)

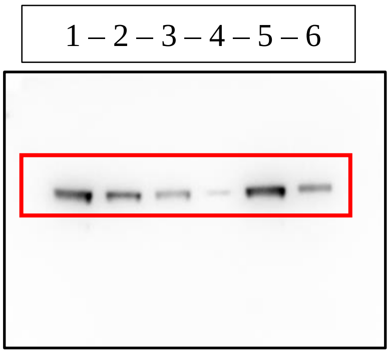

p-AKT (Substantia Nigra)

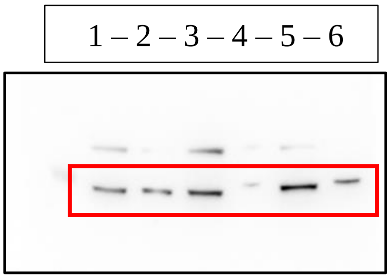

β–Actin (Substantia Nigra)

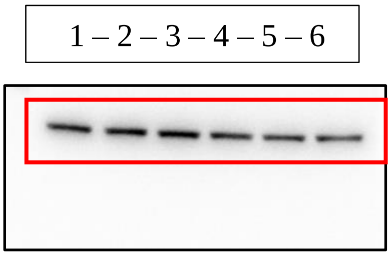

Supplement: Supplementary file 1 — Supplementary Material 1 [file 40659_2023_438_MOESM1_ESM.pdf]
