# Supplementary figures and images for: Neuroprotective effects of the PPARβ/δ antagonist GSK0660 in in vitro and in vivo Parkinson’s disease models
Source: Biol Res. 2023 May 25;56:27. doi: 10.1186/s40659-023-00438-1 (PMC10210307; doi:10.1186/s40659-023-00438-1)

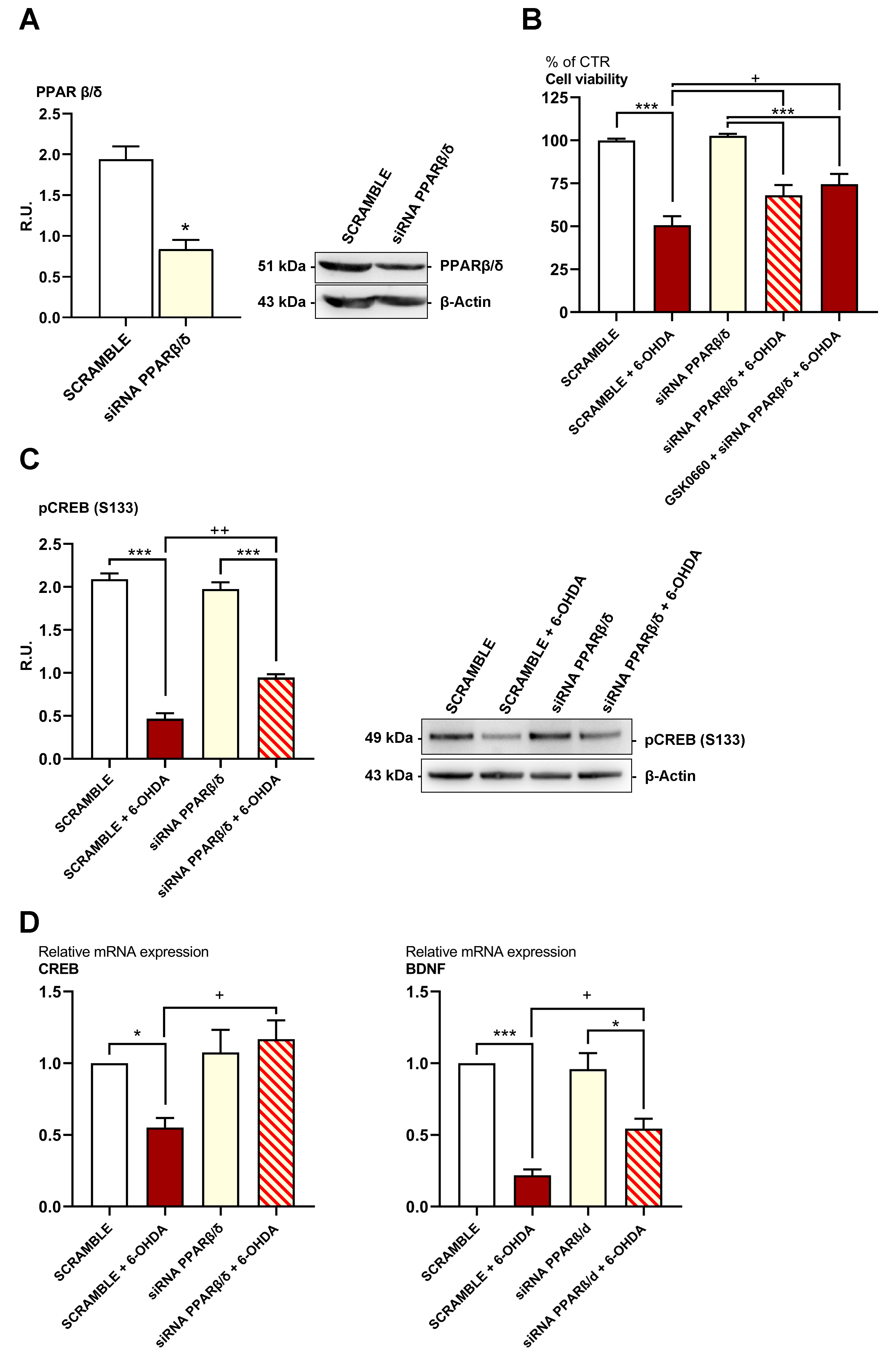

Supplement: Supplementary file 2 — Supplementary Material 2 [file 40659_2023_438_MOESM2_ESM.tif]
